# Biochemical and Comparative Transcriptome Analyses Reveal Key Genes Involved in Major Metabolic Regulation Related to Colored Leaf Formation in *Osmanthus fragrans* ‘Yinbi Shuanghui’ during Development

**DOI:** 10.3390/biom10040549

**Published:** 2020-04-04

**Authors:** Xuan Chen, Xiulian Yang, Jun Xie, Wenjie Ding, Yuli Li, Yuanzheng Yue, Lianggui Wang

**Affiliations:** 1 Key Laboratory of Landscape Architecture, Jiangsu Province, College of Landscape Architecture, Nanjing Forestry University, No. 159 Longpan Road, Nanjing 210037, China; chenxuan@njts.edu.cn (X.C.); xly@njfu.edu.cn (X.Y.); wenjieding@njfu.edu.cn (W.D.); chestnutlyl@njfu.edu.cn (Y.L.); 2 College of Fine Arts, Nanjing Normal University of Special Education, No.1 Shennong Road, Nanjing 210038, China; xiejun@njts.edu.cn; 3 Co-Innovation Center for Sustainable Forestry in Southern China, Nanjing Forestry University, Nanjing 210037, China

**Keywords:** leaf color, transcriptome, chlorophyll metabolism, carotenoid metabolism

## Abstract

*Osmanthus fragrans* ‘Yinbi Shuanghui’ not only has a beautiful shape and fresh floral fragrance, but also rich leaf colors that change, making the tree useful for landscaping. In order to study the mechanisms of color formation in *O. fragrans* ‘Yinbi Shuanghui’ leaves, we analyzed the colored and green leaves at different developmental stages in terms of leaf pigment content, cell structure, and transcriptome data. We found that the chlorophyll content in the colored leaves was lower than that of green leaves throughout development. By analyzing the structure of chloroplasts, the colored leaves demonstrated more stromal lamellae and low numbers of granum thylakoid. However, there was a large number of plastoglobuli. Using transcriptome sequencing, we demonstrated that the expression of differentially expressed genes (DEGs) involved in chlorophyll degradation was upregulated, i.e., heme oxygennase-1 (*HO1)*, pheophorbide a oxidase (*PAO),* and chlorophyllase-2 (*CLH2)*, affecting the synthesis of chlorophyll in colored leaves. The stay-green gene (*SGR*) was upregulated in colored leaves. Genes involved in carotenoid synthesis, i.e., phytoene synthase 1 (*PSY1*) and 1-Deoxyxylulose-5-phosphate synthase (*DXS*), were downregulated in colored leaves, impeding the synthesis of carotenoids. In the later stage of leaf development, the downregulated expression of Golden2-Like (*GLK*) inhibited chloroplast development in colored leaves. Using weighted gene co-expression network analysis (WGCNA) to investigate the correlation between physiological indicators and DEGs, we chose the modules with the highest degree of relevance to chlorophyll degradation and carotenoid metabolism. A total of five genes (*HSFA2*, *NFYC9*, *TCP20*, *WRKY3,* and *WRKY4*) were identified as hub genes. These analyses provide new insights into color formation mechanisms in *O. fragrans* ‘Yinbi Shuanghui’ leaves at the transcriptional level.

## 1. Introduction

Leaf color mutants exist widely in higher plants and are ideal materials for the study of physiological metabolic processes such as plant photosynthesis, photochemical function, phytohormones, and disease resistance mechanisms. These mutants can also be analyzed to identify gene functions and investigate inter-gene interactions. To date, researchers have obtained these mutants from crops such as *Oryza sativa* [1], *Triticum aestivum* [2], *Arabidopsis thaliana* [3], and *Brassica napus* [4]. A large number of studies have been carried out on pigment content metabolism [5], chloroplast development [6], photosynthetic physiology [7], genetic patterns [8], gene cloning [9,10], etc. These studies provide important basic information that aided the understanding of the mechanisms that underlie leaf color mutations and related gene function.

The direct causes of leaf color mutations include the changes in leaf pigment content and proportion, cell structure, and physiological and biochemical metabolism. The mechanism underlying leaf color variation is complex and mainly related to chlorophyll, carotenoid, secondary metabolite synthesis, photosynthesis, and chloroplast development. Remarkably, the leaf color changes in the leaf mutant and senescence process are usually related to the chlorophyll metabolism pathway [11,12]. However, the change in leaf color during leaf senescence is later followed by leaf drop, and the leaf color in coloration mutants start with the initial stage of leaf color formation and would keep this color during leaf growth and development. Studies on leaf color mutants in different varieties of *Camellia sinensis* show that patterns of differentially expressed genes (DEGs) involved in chlorophyll biosynthesis or degradation, carotenoid biosynthesis or degradation, and chloroplast development affect leaf color [13,14]. Compounds related to carotenoid and chlorophyll synthesis have confirmed that the H subunit of magnesium chelatase (CHLH) and β-carotene hydroxylase (BCH) are important to the abnormal leaf color phenotype of the *T. aestivum* yellow-green leaf mutant [15]. Studies on leaf color mutant of *Cymbidium sinense* ‘Dharma’ have demonstrated that the gene encoding key enzyme of chlorophyll degradation is expressed at a higher level, which is consistent with its lower chlorophyll content. It suggests that leaf color variation may be due to the excessive degradation of chlorophyll [16]. In addition, the upregulated expression of genes involved in flavonoid biosynthesis and the downregulation of genes involved in carotenoid biosynthesis may be related to the yellow-leaf phenotype [17]. A previous study has suggested that the color formation mechanism of the yellow leaf mutant of *Lagerstroemia indica* is influenced by chloroplast development and chlorophyll metabolism [18]. Additionally, the expression of polyphenol oxidase (*PPO*) and non-yellow coloring (*NYC/NOL*) may affect chlorophyll biosynthesis in *Ginkgo biloba* yellow-green mutants and promote chlorophyll b degradation to chlorophyll a. The upregulated expression of 15-cis-ζ-carotene isomerase (*Z-ISO*), ζ-carotene desaturase (*ZDS*), and lycopene ε-cyclase (*LCYE*) enhance carotenoid accumulation [19].

*O. fragrans* is one of the top ten traditional ornamental crops native to China. The tree offers not only a beautiful shape and the fresh floral fragrance of traditional sweet osmanthus, but also rich leaf color changes, making it a good material for landscaping [20]. During diversification, many traits have been mutated due to natural hybridization, manual selection, and other environmental factors. Also, the number of varieties is becoming greater. *O. fragrans* is divided into five varieties: the Aurantiacus, Albus, Asiaticus, Luteus, and Color groups [21]. *O. fragrans* ‘Yinbi Shuanghui’ is derived from the Asiaticus group, which has a limbate leaf with a yellow-white margin and green in the middle.

In order to investigate the mechanisms of leaf color formation in ‘Yinbi Shuanghui’, we selected colored and green leaves from the same plant and analyzed leaf pigment contents, cell structures, and transcriptome data at different developmental stages. By comparing transcriptome data, we identified gene expression patterns related to chlorophyll and carotenoid metabolism. Weighted gene co-expression network analysis (WGCNA) was used to analyze the correlation between physiological indicators and DEGs. We then identified hub genes involved in regulating chlorophyll and carotenoid metabolic pathways at different developmental stages. This study provides a reference that could be used to investigate the color formation mechanisms of leaf color mutants.

## 2. Material and Methods

### 2.1. Plant Materials

A healthy adult *O. fragrans* ‘Yinbi Shuanghui’ tree bearing both colored leaves (C) and green leaves (G) was used for transcriptome analysis. The plants were 3 years old and had been grown in the field at Liyang (N 31°43’, E 119°48’), China. ‘Yinbi Shuanghui’ was a colored-leaf cultivar that was evolved from *O. fragrans* ‘Sijigui’. Three developmental stages for leaf color in ‘Yinbi Shuanghui’ were identified: the young leaf stage (A), leaf-expanding stage (B), and mature leaf stage (C). We collected leaf samples from colored and green leaves at three different stages, respectively, to measure the pigment content and cell structure, as well as for transcriptome sequencing analysis. All samples were collected at 10 am, transferred immediately to liquid nitrogen, and stored subsequently at -80 °C until RNA extraction. Leaves at each stage were sampled from three comparable plants using three biological replications. The samples collected at stages A, B, and C were used to construct 18 libraries.

### 2.2. Pigment Determination

Pigments were extracted from leaves using the methods described by Zhang [22]. Chlorophyll and carotenoids were extracted with 95% ethanol for 24 h and quantified spectrophotometrically (Lambda 365, PerkinElmer, USA) at 470, 645, and 663 nm. Anthocyanin was extracted with 10% hydrochloric acid in darkness for 24 h and quantified spectrophotometrically at 530 nm.

### 2.3. Transmission Electron Microscopy

Samples dissected from yellow and green parts of colored leaves and green leaves were cut into smaller sections that were approximately 1.0 mm × 1.0 mm × 1.0 mm in size. The samples were fixed in 4% glutaraldehyde for 24 h at 4 °C, washed twice with 0.1 M phosphate buffer for 10 min each time, then fixed in 1% OsO_4_ for 5 h, and then washed twice with 0.1 M phosphate buffer for 10 min each time. Then dehydration was carried out with acetone of different concentrations of 50%, 60%, 70%, 80%, 90%, and 100% from low to high for 20 min each time. The epoxy resin permeated according to a gradient of volume ratio of 3:1, 3:2, 3:3, each for 2 h, and the pure epoxy resin permeated for 24 h. The samples were placed on the embedding plate and added with epoxy resin. The samples were put in an oven at 45°C for 12 h. The resin was polymerized, taken out and cooled to room temperature. Sections were cut into 50 nm thick using a Leica EMUC6 ultramicrotome (Leica Microsystems GmbH, Wentzler, Germany). Then the sections were stained with uranyl acetate and lead citrate for 30 min. The JEM-1400 transmission electron microscope (JEOL Ltd., Tokyo, Japan) was then used to examined and imaged the ultrastructure. The chloroplasts in 10 randomly selected cells were counted the average number of chloroplasts per cell. The length and width of chloroplasts in 10 intact chloroplasts distributed in different cells were measured by AutoCAD.

### 2.4. RNA Extraction, cDNA Library Preparation, and Sequencing

Total RNA was extracted from the colored and green leaves separately using an RNA Purification Kit (Tiangen Biotech Co., Beijing, China) following the manufacturer’s instructions. Three replicates for each sample were used. The integrity of the RNA was verified by RNase-free agarose gel electrophoresis and the concentration was measured using a Nano Drop 2000 spectrophotometer (Thermo Scientific, Waltham, MA, USA). High-quality RNA from leaves at each stage was mixed in equal quantities for subsequent RNA sequencing. For each developmental stage of colored and green leaves, three RNA samples were used in the construction of a cDNA library and Illumina sequencing, which was completed by Gene Denovo Biotechnology Co. (Guangzhou, China). The transcriptome data has been uploaded to the NCBI Sequence Read Archive (https://www.ncbi.nlm.nih.gov/sra/) under the accession number SRP238684.

### 2.5. De Novo Assembly of RNA-Seq Reads and Quantifying Gene Expression

Transcriptome de novo assembly was carried out in this study. Before assembly, adapter sequences were removed from the raw reads. Then, low-quality reads (with over 40% of bases with quality scores of 10 or lower and/or over 10% of bases unknown) were removed from each data set to establish more reliable results. Following this, clean, high quality reads from all the samples were combined and assembled using Trinity to construct unique consensus sequences for reference. Sequencing reads were remapped to the reference sequences by SOAP aligner/soap2. For each gene, the expression level was measured by Reads Per Kilobase exon Model per Million mapped reads (RPKM), based on the number of uniquely mapped reads, to eliminate the influence of different gene lengths and sequencing discrepancies in the gene expression calculation. For genes with more than one alternative transcript, the longest transcript was selected to calculate the RPKM.

### 2.6. Identification and Functional Analysis of DEGs

To identify genes that were differentially expressed between the libraries created from colored and green leaves, DEGs from different stages were identified by comparing the expression levels at stage A with those at stage B and stage C in the two leaf types, respectively. To correct for multiple testing, the false discovery rate (FDR) was calculated to adjust the threshold P-value. Transcripts with a fold change ≥ 2 and FDR < 0.05 were considered to be differentially expressed between the stages. The DEGs identified were used for Gene Ontology (GO) and Kyoto Encyclopedia of Genes and Genomes (KEGG) enrichment analysis. GO enrichment analysis of all DEGs was implemented using the GO seq R package, based on Wallenius non-central hypergeometric distribution. GO terms were assigned to the up- and downregulated DEGs, with a corrected *P* = 0.01. For pathway enrichment analysis, all DEGs were mapped to pathways in the KEGG database to identify significantly enriched KEGG pathways. DEGs were considered significantly enriched in a metabolic pathway at *q* ≤ 0.05 compared with the whole transcriptome background.

### 2.7. Validation of DEGs by Quantitative Real-Time PCR (qRT-PCR)

To validate the results from RNA-Seq and DGE analysis, 22 DEGs associated with chlorophyll metabolism, photosynthesis, biosynthesis of secondary metabolites, and photosynthesis were selected for qRT-PCR. The specific primers used in the experiment to detect the genes expression levels were designed by Primer Premier 5.0 software (Table S1). The *OfACT* gene was used as an internal control in each qRT-PCR experiment[23]. qRT-PCR was performed using ABI StepOnePlus Systems (Applied Biosystems, Foster city, CA, USA). The RNA samples were quantified using a NanoDrop 2000 spectrophotometer (Thermo Scientific, Waltham, MA, USA) and SYBR Premix Ex Taq (Takara Biotechnology, Dalian, Liaoning Province, China). The cDNAs were synthesized from 5 μg total RNA and diluted 10-fold for gene expression experiments. Relative gene expression levels were calculated according to the 2^−ΔΔCt^ comparative CT method. The qRT-PCR and DGE analysis results were presented as fold changes in gene expression relative to the control samples.

## 3. Results

### 3.1. Phenotypic and Physiological Characterization in Colored and Green Leaves

**Figure 1:**
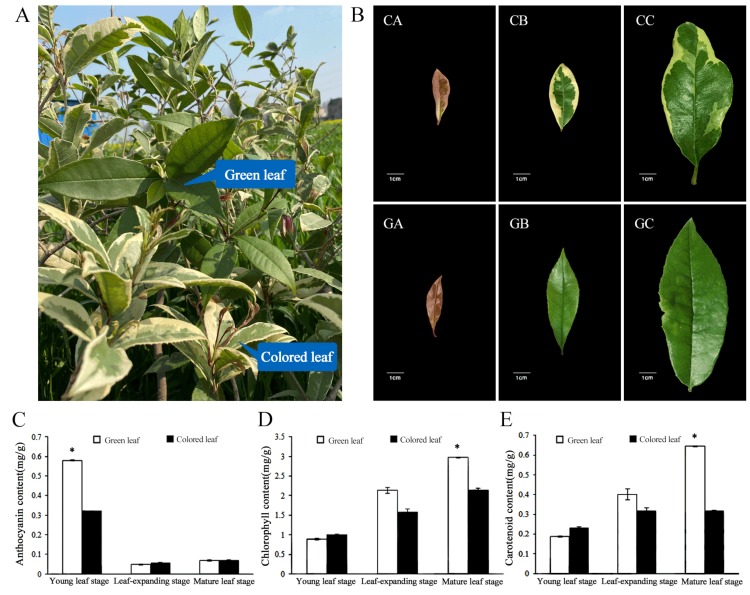
(**A**) Phenotype of *O. fragrans* ‘Yinbi Shuanghui’; (**B**) Phenotype of the colored leaf in young leaf stage (CA), leaf-expanding stage (CB), and mature leaf stage (CC); Phenotype of the green leaf in young leaf stage(GA), leaf-expanding stage (GB), and mature leaf stage (GC); (**C**) Anthocyanin content in colored and green leaf; (**D**) Chlorophyll content in colored and green leaf; (**E**) Carotenoid content in colored and green leaf. The significance of differences compared with green leaf is indicated with an asterisk (*p*< 0.05).

The green leaves of *O. fragrans* ‘Yinbi Shuanghui’ grew on the same plant as colored leaves (Figure 1A). At the earliest stage, the young green leaves appeared dark red, while the young colored leaves were green with a light red edge. The dark red leaves then turned green, while the colored leaves gradually formed a limbate pattern with a yellow-white margin and green middle. Finally, the green area in the middle of the colored leaves gradually expanded and the yellow edge narrowed, but the green leaves remained green (Figure 1B).

To characterize the phenotypic color changes in the leaves, we analyzed the changes in pigment content in the green and colored leaves. The anthocyanin content of green leaves was significantly higher than that of colored leaves at the young leaf stage (Figure 1C). The total chlorophyll and carotenoid contents of the two leaf types increased during leaf development. The content of chlorophyll and carotenoid in green leaves was significantly higher than that in colored leaves at the mature leaf stage (Figure 1D,E). Chl a/Chl b showed significant differences at the young leaf stage and mature leaf stage (Table 1).

**Figure 2:**
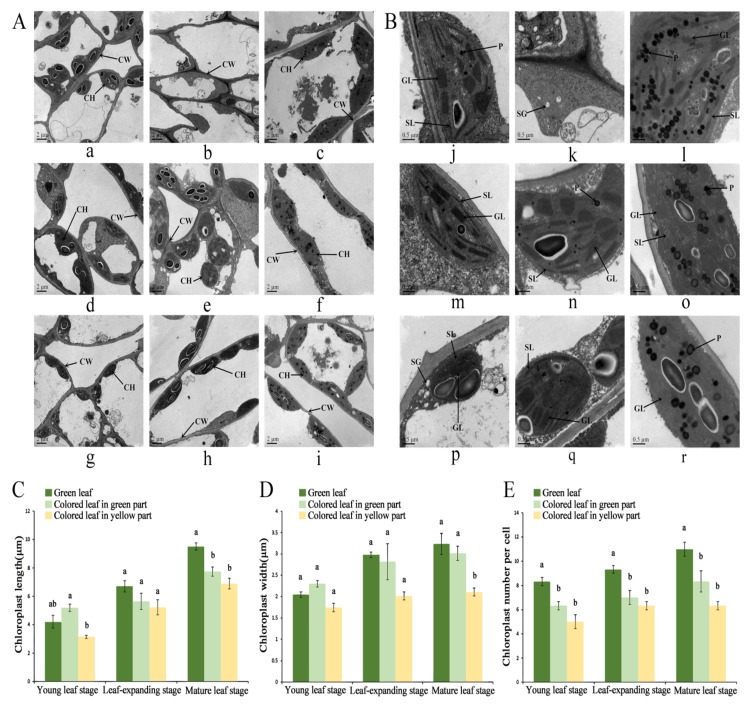
Comparison of ultrastructure of chloroplasts in colored and green leaf. (**A**) Bar = 2 μm (**a**–**i**); (**B**) Bar = 0.5 μm (**j**–**r**); (**C**) Chloroplasts length in colored and green leaf; (**D**) Chloroplasts width in colored and green leaf; (**E**) Chloroplasts number per cell in colored and green leaf. Different letters in the same stage denote significant differences according to Tukey test at the 0.05 level. Chloroplasts ultrastructure of green leaf from young leaf stage to mature leaf stage (**a**–**c**,**j**–**l**); Chloroplasts ultrastructure of colored leaf in green part from young leaf stage to mature leaf stage (**d**–**f**,**m**–**o**); Chloroplasts ultrastructure of colored leaf in yellow part from young leaf stage to mature leaf stage (**g**–**i**,**p**–**r**); CH: chloroplasts; CW: cell wall; GL: granum thylakoid; SL: stromal lamellae; SG: starch grain; P: plastoglobuli.

We further compared the ultrastructure of the chloroplasts in green leaves and the green and yellow parts of colored leaves. In the mesophyll cells of the green leaves (Figure 2A(a–c)), chloroplasts developed typical structures, with a typical granum thylakoid structure consisting of thylakoids, numerous starch grains, and few plastoglobuli. There were few chloroplasts in the green parts of the colored leaves and their development was seen to be abnormal (Figure 2A(d–f)). There was no obvious membrane structure and a small number of granum thylakoids and plastoglobuli. In the yellow part of the colored leaf (Figure 2A(g–i)), there were more stromal lamellae and the thylakoids were not normal. The number of granum thylakoids was low, but there was a large number of plastoglobuli. The number of chloroplasts in green leaves was significantly higher than that in colored leaves. There were significant differences in chloroplast length and width between green leaves and yellow parts of colored leaves at the mature leaf stage (Figure 2C–E). The results indicated that the changes in chloroplast structure in the colored leaves affected the pigment distribution.

### 3.2. Transcriptome Analysis

A total of 18 samples of colored and green leaves from different developmental stages of *O. fragrans* ‘Yinbi Shuanghui’ were sequenced. After removing the low mass and linker sequences, 18 samples provided 755,544,800 clean reads with a total of 117,890,827,944 bases. The values for each sample were shown in Table 2. The percentage of Q20 was 95% or more. The GC content of each sample ranged from 47.43-45.75%. The ratio of clean reads from each sample ranged from 94.01-97.79%. The mapping ratio, which was compared with the reference sequence, ranged from 88.46-86.11% (Table 3). The concentration of the three biological repeats was relatively concentrated, indicating that there was good reproducibility between the repeats and sequencing results (Figure S1).

### 3.3. Identification and Functional Annotation of DEGs during Leaf Development

**Figure 3:**
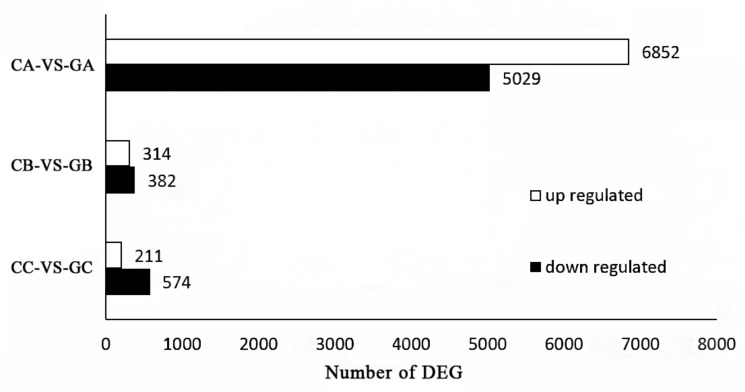
DEGs in colored and green leaves. The colored leaf in young leaf stage (CA), leaf-expanding stage (CB), and mature leaf stage (CC); the green leaf in young leaf stage (GA), leaf-expanding stage (GB), and mature leaf stage (GC).

To identify variations in gene expression between the colored and green leaves at different stages, we used the RPKM values to calculate the DEGs. The DEGs were identified and filtered using the following criteria: *p* < 0.05 and log_2_ (fold change) > 1. We compared the DEGs in the colored and green leaves during leaf development (Figure 3). The volcano plots map was constructed with DEGs to show the expression patterns of all the samples at different stages (Figure S2). In the young leaf stage, there were 11,881 unique DEGs in the two leaf types and the DEGs were mainly upregulated: 6852 were upregulated and 5029 were downregulated. In the leaf-expanding stage, there were 696 DEGs. Among these, there were 314 upregulated and 382 downregulated genes. At the mature leaf stage, there were 785 DEGs, of which 211 were upregulated and 574 were downregulated.

**Figure 4:**
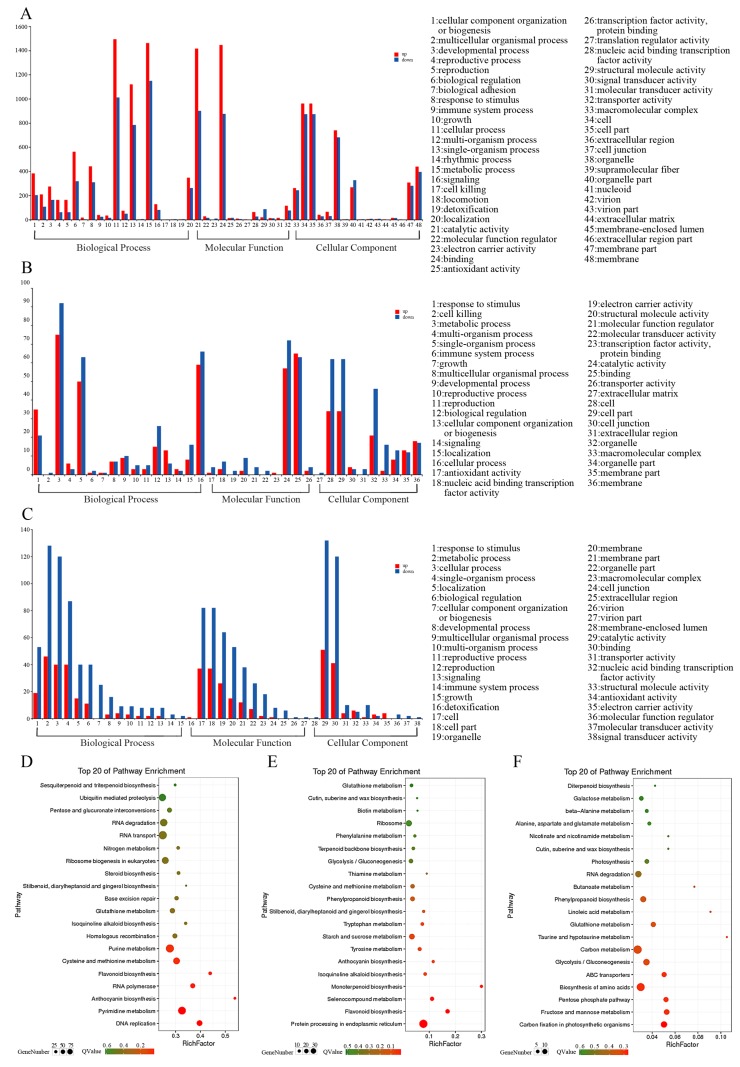
Gene Ontology (GO) classification of unigenes and Top 20 enriched KEGG pathway among the annotated DEGs of colored and green leaf in different leaf stages. (**A**,**D**) CA-VS-GA; (**B**,**E**) CB-VS-GB; (**C**,**F**) CC-VS-GC. The colored leaf in young leaf stage (CA), leaf-expanding stage (CB), and mature leaf stage (CC); the green leaf in young leaf stage (GA), leaf-expanding stage (GB), and mature leaf stage (GC)**.**

The functions of these DEGs were classified according to the GO database using the Blast2GO software suite. GO analysis found that DEGs in colored and green leaves were mainly concentrated in ‘molecular function’, ‘cell composition’, and ‘biological processes’. For the colored and green leaves in the young leaf stage (Figure 4A), the GO term of the upregulated DEGs was significantly enriched in cellular processes (1495, 21.82%), metabolic processes (1464, 21.37%), and binding (1448, 21.13). Downregulated DEGs were significantly enriched in binding (1448, 21.13%), catalytic activity (1417, 20.68%), and metabolic processes (1151, 22.89%). In the leaf-expanding stage (Figure 4B), the GO terms for the upregulated DEGs in colored and green leaves were significantly enriched in metabolic processes (75, 23.89%), binding (65, 20.70%), and cellular processes (59, 18.79%), whereas the downregulated DEGs were significantly enriched in metabolic processes (92, 24.08%), catalytic activity (72, 18.85%), and cellular processes (66, 17.28%). During the mature leaf stage (Figure 4C), the GO term of the upregulated DEGs was significantly enriched in catalytic activity (51, 24.17%), metabolic processes (46, 21.80%), and binding (41, 19.43%). However, the downregulated DEGs were significantly enriched in catalytic activity (132, 23.00%), metabolic processes (128, 22.30%), cellular processes (120, 20.91%), and combinations (120, 20.91%).

There were significant differences in the KEGG metabolic pathways enriched by DEGs in colored and green leaves at different stages. In the young leaf stage, there were 1915 DEGs with pathway annotation in colored and green leaves. The highest expressed DEG was the gene encoding the ribosomal protein (Table S2). These DEGs were enriched in 127 KEGG pathways and the number of DEGs enriched in each pathway was different (Figure 4D). Six of the pathways were significantly enriched (Table S3). DNA replication was the most significant enrichment pathway, followed by the pyrimidine metabolic pathway, anthocyanin biosynthetic pathway, RNA polymerase pathway, and flavonoid biosynthesis pathway. Among these, the upregulated genes in colored leaves associated with leaf color expression were significantly enriched in the anthocyanin biosynthesis and flavonoid biosynthesis pathways, with 14 and 18 DEGs, respectively. Significant downregulation of genes in colored leaves was seen in the photosynthesis pathway.

During the leaf-expanding stage, we performed a pathway analysis of 147 DEGs in colored and green leaves. The highest expressed DEGs were the genes encoding ribosomal protein and cytochrome c oxidase (Table S2). Ribosomal protein played an important role in the translation of key proteins involved in chloroplast development and photosynthesis [24]. A decrease in cytochrome c oxidase activity produced strong alterations of plant growth and development [25]. Our study found that the genes encoding ribosomal protein and cytochrome c oxidase were downregulated in colored leaves. The downregulated expression of these genes may be responsible for the change in leaf color. As shown, the DEGs were enriched in 72 KEGG pathways (Figure 4E). Four of the pathways were significantly enriched (Table S4). These were the protein processing pathway in the endoplasmic reticulum, flavonoid biosynthesis pathway, organic selenium-containing compound metabolic pathway, and monoterpenoid biosynthetic pathway. In colored leaves, the upregulated genes associated with leaf color expression were significantly enriched in anthocyanin biosynthesis and flavonoid biosynthesis, whereas the pathway in which the downregulated genes were significantly enriched was that for phenylpropanoid biosynthesis.

There were 141 DEGs in the colored and green leaves at the mature leaf stage, which were enriched in 76 KEGG pathways (Figure 4F). The highest expressed DEGs were the genes encoding crystal protein and camp-regulated d2 protein (Table S2). Only the biosynthesis of amino acid pathway was significantly enriched (Table S5). However, there was no significantly enriched pathways associated with leaf color expression.

### 3.4. Identification of DEGs Related to Pigment Metabolism

#### 3.4.1. Identification of DEGs Related to Chlorophyll Metabolism

**Figure 5:**
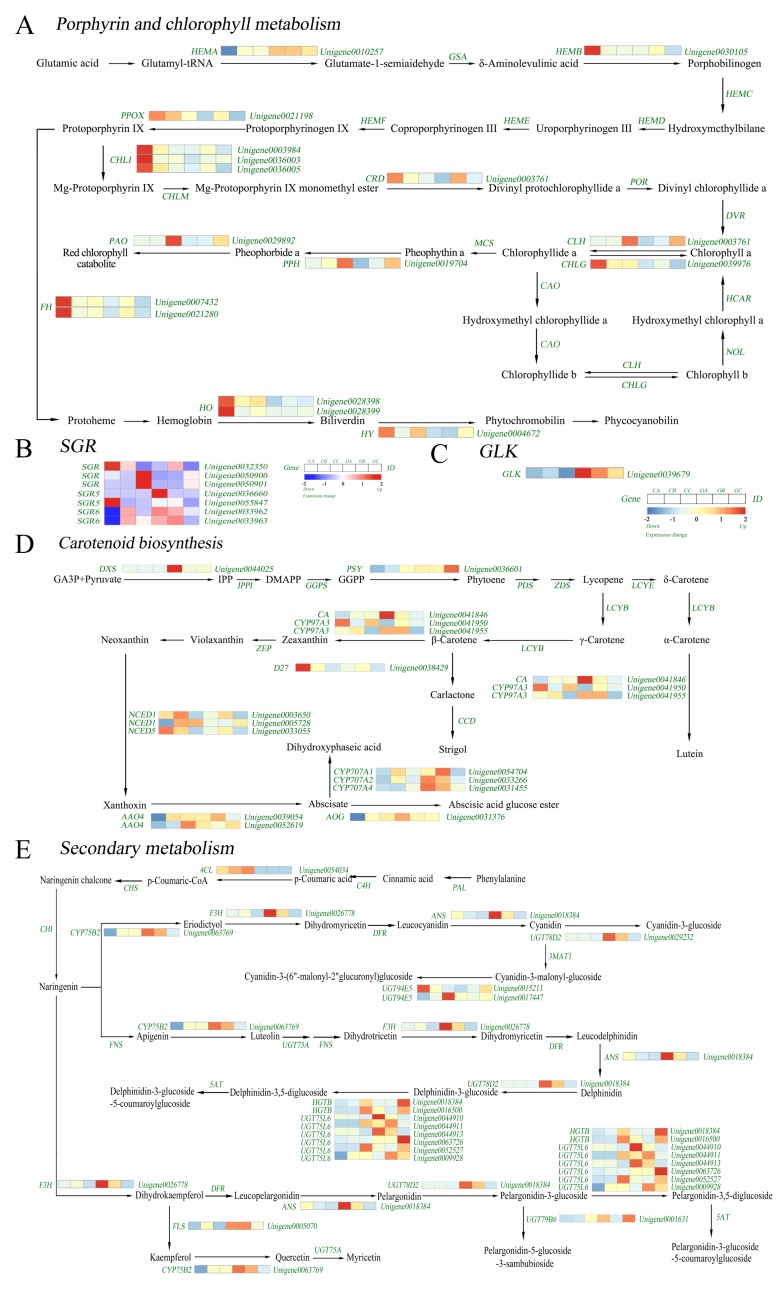
(**A**) Expression profiles of differentially expressed genes (DEGs) involved in porphyrin and chlorophyll metabolism between colored and green leaf; (**B**) Expression profiles of DEGs involved in *SGR* between colored and green leaf; (**C**) Expression profiles of DEG involved in *GLK* between colored and green leaf; (**D**) Expression profiles of DEGs involved in carotenoid biosynthesis between colored and green leaf; (**E**) Expression profiles of DEGs involved in biosynthesis of secondary metabolites between colored and green leaf.

Among all the annotated pathways, we focused on pathways closely related to pigment synthesis, such as the chlorophyll, carotenoid, and secondary metabolic pathways. To better understand the chlorophyll metabolic in green and colored leaves, the enzymes involved in the porphyrin and chlorophyll metabolism pathways were investigated, and the expression of genes encoding these enzymes was significantly different. (Figure 5A). We found that there were DEGs with significant differences in the chlorophyll metabolism pathway at the young leaf stage and the differences in DEGs between the leaf-expanding and mature leaf stages were not significant. During the young leaf stage, 16 genes were found to annotate 11 enzymes in the porphyrin and chlorophyll metabolic pathways. There were 11 DEGs involved in chlorophyll synthesis. Unigene0010257, encoding Glutamyl t-RNA reductase (*HEMA1*), was significantly upregulated during the young leaf stage in green leaves and its expression was generally upregulated during all three stages. There were also five DEGs involved in chlorophyll degradation, including Unigene0028398 and Unigene0028399, encoding heme oxygennase-1 (*HO1*), Unigene0029892, encoding pheophorbide a oxidase (*PAO*), Unigene0017904, encoding pheophytin hydrolase (*PPH*), and Unigene0010601, encoding chlorophyllase-2 (*CLH2*). They were significantly downregulated in green leaves compared with those in colored leaves at the young leaf stage. PAO and CLH2 were key regulatory enzymes in the chlorophyll degradation pathway. The results indicated that the downregulated expression of the coding genes was associated with lower chlorophyll content in the colored leaves.

Overexpression of the *SGR* can promote the decomposition of chlorophyll and reduce the number of granum thylakoids and chlorophyll content in normally-growing leaves. In this study, we identified seven DEGs associated with the *SGR*, based on KEGG pathway enrichment analysis (Figure 5B). Six *SGR* were significantly upregulated in colored leaves during the young leaf stage. At the mature leaf stage, Unigene0050901 encoding *SGR*, showed the same trend and was significantly upregulated in the colored leaves compared with those in green leaves.

Plant photosynthesis was mainly carried out in chloroplasts. The coloration mechanism of plant leaves was closely related to the development and distribution of chloroplasts. In our study, Unigene0039679, which encoded the *GLK* transcription factor associated with chloroplast development, was downregulated in the three stages of colored leaves and significantly downregulated compared with those in green leaves at the young leaf stage (Figure 5C). This indicated that the chloroplast structure of the colored leaves was different from that of the green leaves in the early stage of leaf development.

#### 3.4.2. Identification of DEGs Related to Carotenoid Metabolism

In the young leaf stage, we determined that 16 DEGs with significant differences in the carotenoid pathway. Unigene0036601, encoding phytoene synthase 1 (*PSY1*), and Unigene0044025, encoding 1-Deoxyxylulose-5-phosphate synthase (*DXS*), were related to carotenoid synthesis, were significantly downregulated in colored leaves (Figure 5D). Among them, Unigene0044025 was significantly downregulated during the three developmental stages of colored leaves. However, Unigene0033055, encoding 9-cis-epoxycarotenoid dioxygenase (*NCED*), related to carotene degradation, was significantly upregulated in colored leaves. These results indicated that the expression level of the genes related to carotenoid synthesis in colored leaves was lower than that in green leaves.

#### 3.4.3. Identification of DEGs Related to Secondary Metabolism

In this study, 6 DEGs related to the flavonoid synthesis pathway were found (Figure 5E). In the young leaf stage, the expression of Unigene0026778 encoding flavanone 3-hydroxylase (*F3H*), and Unigene0018384 encoding anthocyanidin synthase (*ANS*) were significantly lower in the colored leaves than in the green leaves. Further analysis of the anthocyanin biosynthesis pathway revealed 14 DEGs. Six genes encoding UDP-glucosyltransferase (*UGT75L6*) were significantly different in the mature leaf stage of green leaves and colored leaves. The expression of Unigene0015211 encoding UDP-glucosyltransferase (*UGT94E5*) was higher in the colored leaves of the three stages than in the green leaves. The above results indicated that the upregulated expression of genes involved in the secondary metabolic pathway might be related to the formation of colored leaves at the young leaf stage.

### 3.5. Identification of DEGs Co-Expression Modules by WGCNA

RNA-seq sequencing was used to analyze the DEGs of colored and green leaves. At the same time, WGCNA was used to analyze the association between the co-expressed gene modules formed by DEGs and physiological indicators. Studies had found that the photosynthetic pigment content of colored leaf plant can affect photosynthetic performance [26]. In this study, the pigment content and parameters of photosynthesis and fluorescence in green and colored leaves were measured as physiological indicators. Low levels of gene expression were filtered out by data analysis to obtain 39,965 highly expressed genes. The gene clustering tree was constructed according to the correlation between the expression levels of genes and the gene module. The modules were divided according to the clustering relationship between genes. The modules with similar expression patterns were then merged according to the similarity of the module feature values (Figure S3). By screening the weight values, a total of 15 co-expression modules were obtained. The lavenderblush1 module contained the largest number of genes, 9213 genes, and the Navajo white module contained the lowest number of genes, 88 genes.

**Figure 6:**
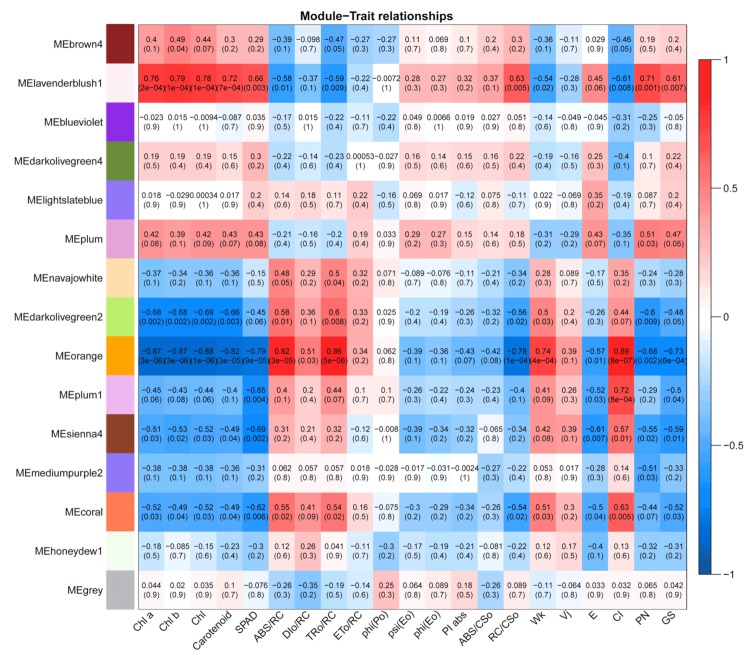
Trait-module associated heat map. Each column represents a physiological indicator, and each row represents a genetic module. The number in each grid represents the correlation between the module and the trait. The number in parentheses represents the significance P-value. The smaller the P-value, the stronger the significance of the representativeness and module correlation. Chl a: content of chlorophyll a; Chl b: content of chlorophyll b; Chl: content of chlorophyll; Carotenoid: content of carotenoid; SPAD: chlorophyll relative value; ABS/RC: photon absorbance rate per active reaction center; DIo/RC: exciton dissipation rate per active reaction center; TRo/RC: exciton trapping rate per active reaction center; ETo/RC: electron transport per active reaction center; phi(Po): maximum efficiency of PSII photochemistry; psi(Eo): the efficiency with which a trapped exciton can move an electron into electron transport chain further than QA; phi(Eo): the quantum yield of electron transport; PI abs: performance index on absorption basis.; ABS/CSo: photon absorbance per excited cross-section; RC/CSo: number of reaction centers per excited cross-section; Wk: the ratio of variable fluorescence Fk to amplitude Fo-Fj; Vj: the ratio of variable fluorescence Fj to amplitude Fo-Fp; E: transpiration rate; CI: intercellular; CO_2_; PN: photosynthetic rate; GS: stomatal conductivity.

By calculating the correlation between the eigenvalues and traits of each module, we found that the ME display of the orange module and the lavenderblush1 module was highly correlated with the physiological indicators of leaf coloration. Chlorophyll and carotenoid metabolism were positively correlated with the lavenderblush1 module and negatively correlated with the orange module (Figure 6). This indicated that the lavenderblush1 module might play an important role in colored leaves, whereas the orange module may play an inhibitory role. In analyzing the enrichment of chlorophyll metabolism- and carotenoid metabolism-related genes, we found that the coral module contained the largest number of DEGs involved in chlorophyll metabolism, but only one DEG related to carotenoid metabolism. The lavenderblush1 module contained 14 DEGs related to the chlorophyll synthesis pathway and 14 DEGs associated with carotenoid metabolism (Table 4). We speculate that the lavenderblush1 module may be related to the color formation mechanism.

**Figure 7:**
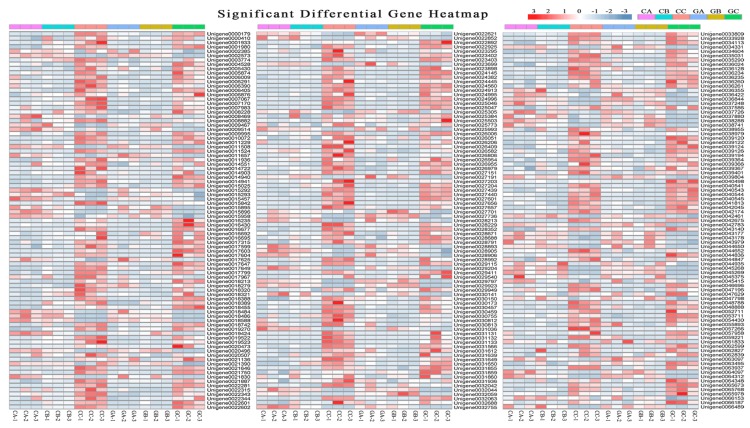
Heatmap of transcription factors in the lavenderblush1 module. The colored leaf in young leaf stage (CA), leaf-expanding stage (CB), and mature leaf stage (CC); the green leaf in young leaf stage (GA), leaf-expanding stage (GB), and mature leaf stage (GC); 1–3: Three biological replicates of each leaf sample.

To systematically study the regulation of chlorophyll and carotenoid metabolism, we extracted transcription factors from the lavenderblush1 module. The lavenderblush1 module had 261 transcription factors belonging to 52 transcription factor families (Table S6). The main transcription factor families were *WRKY* (41 unigenes), *bHLH* (30 unigenes), and *ERF* (26 unigenes) (Figure S4). Figure 7 showed that these transcription factors exhibited different expression patterns. Cluster analysis among three biological repeats showed that the expression patterns of transcription factors in green and colored leaves were different during the development stage (Figure S5).

### 3.6. Co-Expression Networks Reveal a Differential Regulatory Network of Chlorophyll Metabolism and Carotenoid Metabolism

**Figure 8:**
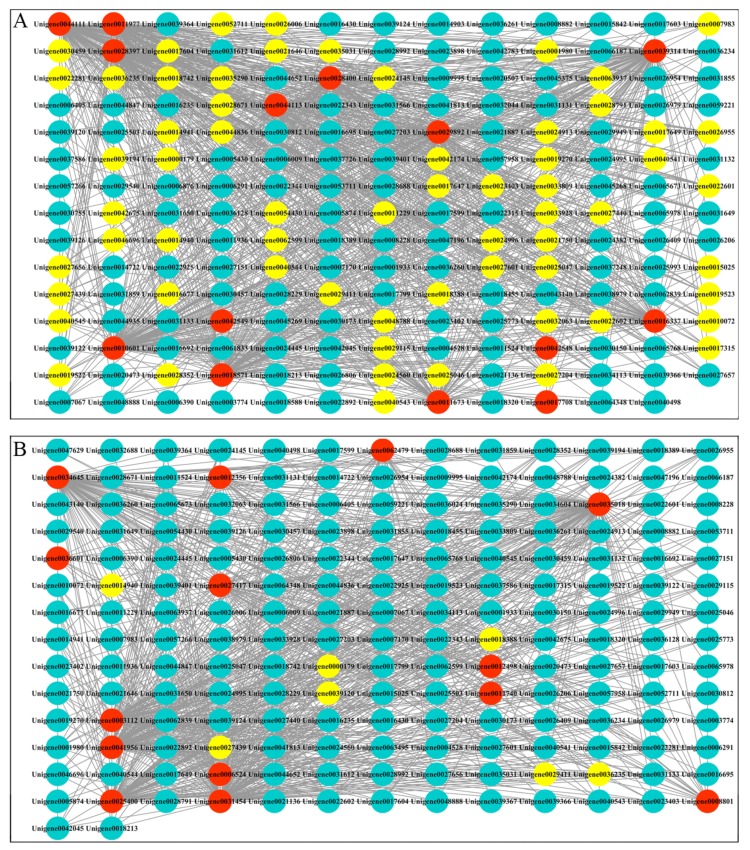
(**A**) Construction of regulatory networks of chlorophyll metabolism gene and transcription factors in the lavenderblush1 module; (**B**) Construction of regulatory networks of carotenoid metabolism gene and transcription factors in the lavenderblush1 module. Red nodes indicate structural genes, blue nodes indicate transcription factors, yellow nodes indicate transcription factors with edges greater than 10.

To understand the regulatory networks of chlorophyll and carotenoid metabolism genes with transcription factors, we first screened 28 related genes in the lavenderblush1 module (Table S7). Then we selected 261 transcription factors from the lavenderblush1 module for Pearson correlation analysis. We plotted all pairs of adjustment relationships using a Pearson correlation coefficient threshold greater than 0.7 (Figure 8). The visualization in Cytoscape showed that there were 180 nodes connected to 1226 edges in the chlorophyll metabolism regulatory networks. There were 170 nodes connected to 922 edges in the carotenoid metabolism regulatory networks. Based on an edge greater than 10, we obtained 5 candidate hub genes for the regulation of chlorophyll and carotenoid metabolism (Table 5).

### 3.7. Verification of the Gene Expression through qRT-PCR

**Figure 9:**
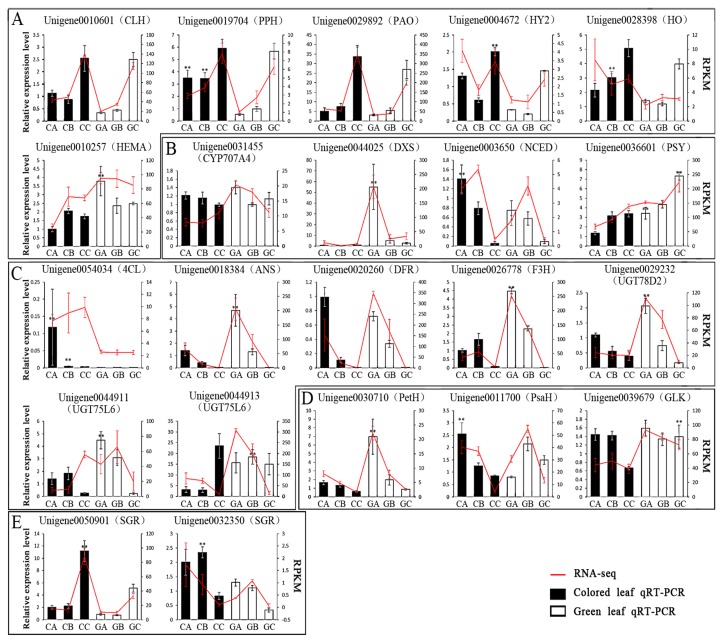
qRT-PCR validation of transcriptome data results for genes associated with leaf color changes. (**A**) DEGs involved in Porphyrin and chlorophyll metabolism; (**B**) DEGs involved in Carotenoid biosynthesis; (**C**) DEGs involved in Biosynthesis of secondary metabolites; (**D**) DEGs involved in Photosynthesis; (**E**) DEGs involved in *SGR*. The significance of differences compared with green leaf is indicated with two asterisks (*p* < 0.01).

In order to further verify the reliability of the transcriptome data, 22 DEGs related to chlorophyll synthesis, photosynthesis, flavonoid synthesis, anthocyanin synthesis, and carotenoid synthesis were selected for qRT-PCR (Figure 9). The expression patterns of 19 genes detected by qRT-PCR were consistent with the transcriptome expression trend during the three stages of leaf color development but Unigene0032350, encoding *SGR*, Unigene0020260, encoding dihydroflavonol 4-reductase (*DFR*), and Unigene0044913, encoding *UGT75L6*, showed different expression trends. The above disparities may have been caused by differences between transcriptome sequencing and qRT-PCR as detection methods, a certain degree of inconsistency (about 30-40%). The result was normal and reasonable. There was no significant change in gene expression level, resulting in inconsistent detection results. The results showed that the qRT-PCR results of most of the genes were consistent with the transcriptome data.

## 4. Discussion

### 4.1. Colored Leaves are Closely Related to Chloroplast Dysplasia

A decrease in the number of chloroplasts and their imperfect development in leaves affect the synthesis of chlorophyll and change leaf color. During the yellowing of *Ginkgo biloba* leaves, the chloroplast thylakoid membrane layer gradually loosed until the membrane structure gradually disintegrated. The oil particles in the chloroplast increased and eventually disintegrated [27]. The ultrastructure of the colored leaves of *Cymbidium sinense* [16] and *G. biloba* [19] mutants was different from that of normal leaves, exhibiting a relatively low number of chloroplasts. The granum thylakoids had a low degree of overlap and loose arrangement, and the membrane structure was fuzzy. The shape was irregular, and the layers were degraded. There were many plastoglobuli but almost no starch grains. The chloroplast ultrastructure of the yellow leaves of *Lagerstroemia indica* exhibited a ruptured thylakoid membrane and insignificant stromal lamellae [18]. Under natural conditions, the chloroplast granum thylakoids of *Camellia sinensis* ‘Anji Baicha’ were poorly developed. The degradation of granum thylakoids, membrane structure, and lamellar structure hindered the biosynthesis of chlorophyll and carotenoids [12].

The results of this study showed that the chloroplast structure of colored and green leaves in *O. fragrans* ‘Yinbi Shuanghui’ was also significantly different. During the young leaf stage, the chloroplast volume in colored leaves was smaller than that in green leaves. During the mature leaf stage, there were more stromal lamellae in the yellow part of the colored leaves, the granum thylakoids were not stacked normally, and there were a large number of plastoglobuli. The green part of the colored leaf was narrow and small, and the chloroplasts were irregularly long fusiform in shape. The chloroplasts had an unclear outline and no obvious membrane structure. The differences in the structure of these chloroplasts may affect development and photosynthesis in ‘Yinbi Shuanghui’ leaves, resulting in the inhibition of chlorophyll synthesis and formation of colored leaves.

Chloroplasts use light energy and convert it into chemical energy. *GLK*, first discovered in *Zea mays,* was an important transcription factor that affected the development of chloroplasts [28]. Studies in *A. thaliana* [29], *O. sativa* [30] and *Lycopersicon esculentum* [31] found that the expression level of *GLK* affected the expression of photosynthetic genes and the development of chloroplasts. As in rice, *AtGLK1* and *AtGLK2* were expressed in partially overlapping domains in photosynthetic tissue. Insertion mutants demonstrate that this expression pattern reflected a degree of functional redundancy, as single mutants display normal phenotypes in most photosynthetic tissues. However, double mutants were pale green in all photosynthetic tissues and chloroplasts exhibited a reduction in granum thylakoids[30] The leaf color mutant of *Anthurium andraeanum* was accompanied by the downregulation of *GLK* expression. Ultrastructural observations revealed that chloroplast development was prevented, and the structure was incomplete [32]. The *GLK* that regulates chloroplast development in the yellow leaves of *G. biloba* also exhibited downregulated expression [19]. Similarly, the *GLK* expression in the colored leaves was lower than that of green leaves, and further qRT-PCR also confirmed this result. Consistent with previous studies, the green part of the colored leaf in this study showed few chloroplasts with abnormal development and there were a small number of granum thylakoids and plastoglobuli. This suggests that leaf color variation is closely related to chloroplast dysplasia.

### 4.2. Colored Leaves are Affected by the Expression of Genes Related to Chlorophyll Degradation

Leaf color is affected internally by genetics and externally by environmental factors. It is regulated by leaf cell microstructure and physiological and biochemical metabolic levels. In this study, the colored leaves had a yellow-white margin and green center. Changes in the chlorophyll, carotenoid, and anthocyanin contents of leaves are the main causes of leaf color change. By measuring the chlorophyll and carotenoid contents of leaves in this study, we found that the total chlorophyll and carotenoid content of the colored leaves were significantly lower than that of the green leaves, which led to the phenotypic variation seen in the colored leaves.

Chlorophyll is an important pigment involved in chloroplast-based photosynthesis. Its function is to capture light energy and drive electron transfer to the reaction center. The entire chlorophyll biosynthesis process requires a 15-step reaction involving 15 enzymes. A number of genes (27 in total) encoding these enzymes have been isolated in the model plant *A. thaliana* [33]. In the chlorophyll biosynthesis pathway, mutations in the coding genes for anyone enzyme may hinder the synthesis of chlorophyll.

First, L-glutamyl-tRNA is converted to δ-aminolevulinic acid (ALA) in a reaction catalyzed by glutamyl-tRNA reductase (GluTR) and glutamate-1-semialdehyde 2,1 aminomutase (GSA). The genes encoding these two enzymes are *HEMA* and *HEML*, respectively. ALA is an important precursor substance for chlorophyll synthesis. ALA is converted to porphobilinogen (PBG) in a reaction catalyzed by δ-aminolevulinic acid dehydratase, and then 5-step biochemical reactions produce protoporphyrin IX (ProtoIX). Therefore, *HEMA* is a key enzyme gene-regulating chlorophyll synthesis [34] and three *HEMA* genes have been isolated from *A. thaliana*, *HEMA1*, *HEMA2*, and *HEMA3* [35]. Studies have found that the contents of GluTR, ALA, and chlorophyll were all decreased in *A. thaliana* under *HEMA1* gene silencing [36]. In *Lagerstroemia indica* [18] leaf color mutants, *HEMA* transcription levels were reduced, with a concurrent decrease in chlorophyll content. In our study, *HEMA* was significantly downregulated in colored leaves during the young leaf stage and similar results were obtained in qRT-PCR experiments. At the same time, the chlorophyll content in the colored leaves was reduced by 40% compared with that of the green leaves. Studies indicated that the low expression of *HEMA*, a key enzyme gene in the chlorophyll synthesis pathway, caused a decrease in enzyme activity and affected chlorophyll synthesis.

Changes in any gene activity during chlorophyll synthesis and degradation may affect the efficiency of these processes. The investigation of chlorophyll degradation in this study focused on the formation of chlorophyllide a (Chlide a) under the action of CLH, followed by a non-enzymatic reaction involving metal-chelating substance (MCS) to remove Mg^2+^ and form pheophorbide a (Pheide a). The porphyrin ring of Pheide is opened and red chlorophyll metabolite (RCC) produced under the action of PAO. Red chlorophyll metabolite reductase (RCCR) converts it into a primary fluorescent chlorophyll metabolite (pFCC). Finally, non-fluorescent chlorophyll metabolites (NCCs) are produced [35]. CLH has long been recognized as the first enzyme in the chlorophyll degradation pathway. It was first confirmed in *Citrus reticulata*, and then *AtCLH1* and *AtCLH2* were isolated from *A. thaliana* [37]. Harpaz-Saad [38] have suggested that CLH, which catalyzes the hydrolysis of chlorophyll to produce Chlide a, is the rate-limiting enzyme during chlorophyll degradation. In the present study, *CLH2* in the colored leaves was significantly upregulated during the young leaf stage. Additionally, the ratio of chlorophyll a to chlorophyll b in the colored leaves was higher than that of the green leaves during the three stages. Therefore, we conclude that the upregulated expression of *CLH2* in colored leaves promotes the decomposition of chlorophyll. However, there are other studies that disagree. Following the deletion of two chlorophyllase genes in *A. thaliana*, leaves were found to senesce normally. It indicated that *AtCHL1* and *AtCHL2* did not participate in the degradation pathway of chlorophyll [39]. Inhibition of *AtCLH1* expression leads to a reduction in the chlorophyll a/b ratio and inhibition of CLH activity but does not affect the rate of chlorophyll degradation during leaf senescence. CLH may, therefore, play a role in the process of converting chlorophyll b at the initial stage of leaf senescence [40]. Therefore, the role of CLH in the chlorophyll degradation pathway remains to be further studied.

In recent years, it has been found that in another pathway of chlorophyll degradation. Chlorophyll a first removes Mg^2+^ under the action of MCS. The resulting pheophytin a (Phein a) is then removed from the substrate by PPH to form Pheide a[41]. As the amount of *PPH* expression increased, the rate of Phein a conversion to Pheide a is accelerated, thereby increasing the rate of chlorophyll degradation. Gene expression studies of *CLH* and *PPH* during senescence of *Brassica oleracea* found that cytokinin treatment inhibited the expression of *PPH*, which delayed the degradation of chlorophyll [42]. A small amount of Phein a accumulation was found in the colored leaves of the *O. saliva* mutant nyc3 and confirmed the presence of the *PPH* [43]. In this study, Unigene0019704 encoding *PPH* in the colored leaves was upregulated during the three developmental stages. Therefore, we conclude that *PPH* promoted the degradation of chlorophyll in colored leaves, causing changes in the activity of related enzymes. It may affect the inhibition of chlorophyll synthesis and lead to leaf color variation.

Another key enzyme for chlorophyll degradation is PAO, which catalyzes the decomposition of Phein a to form a colorless substance [44]. Our study showed that during the mature leaf stage, the expression of Unigene0029892, encoding *PAO*, in colored leaves was significantly higher than that in green leaves. Its pattern of expression during leaf color development exhibited an increase with the highest level of expression seen at the mature leaf stage. The slowly increasing expression of *PAO* in green leaves concurred with the high chlorophyll content in the lavenderblush1 module seen in WGCNA. Previous studies have reported that during the ripening of *Ficus carica*, the expression of *PAO* was significantly negatively correlated with chlorophyll levels. Its high level of expression in yellow leaves, therefore, indicated a key role of *PAO* in degrading chlorophyll in *F. carica* [45]. The dark induction of senescence in *A. thaliana* leaves showed upregulation of *PAO* expression and protein levels, and *PAO* was thought to regulate chlorophyll degradation mainly through its transcription levels [46]. Therefore, the accumulation of high levels of *PAO* in the mature leaf stage of colored leaves may be the cause of the leaf color phenotype.

The discovery of *SGR* was a landmark in the study of plant chlorophyll degradation mechanisms. Three SGR homologous proteins, SGR1, SGR2, and SGR-LIKE, were found in the chloroplast vesicles of *A. thaliana* but their roles in chlorophyll degradation were different [47,48,49]. SGR1 interacts with LHCII protein and CCEs to form an SGR1-CCE-LHCII macromolecular complex that converts Chl to pFCC, thereby accelerating chlorophyll degradation [48]. In natural senescence and dark induction, an *SGR1-1* mutant displays a stay-green phenomenon and in *SGR1* overexpressing plants, the leaves show yellowing. Both *SGR1* and *SGRL* show specificity in expression. *SGR1* (*At4g2290*) is significantly upregulated during leaf senescence; expression increased rapidly in senescent leaves with a slightly faded green color [49] and the expression of *SGR* in yellow-leaf Orchid varieties is higher than that in green-leaf varieties [17]. In the present study, seven *SGR* DEGs were identified. Unigene0050901 encoding *SGR* was upregulated in colored leaves and most highly expressed during the mature leaf stage, which is consistent with previous studies. We conclude that *SGR* has a regulatory effect on chlorophyll degradation, resulting in blockage of chlorophyll synthesis.

Transcription factors play an important role in regulating chlorophyll metabolism [50]. In *A. thaliana*, *ANAC046*, *EIN3*, and *ORE1* affect chlorophyll degradation by regulating the expression of key genes *NYE1*, *NYC1*, and *PAO* in the chlorophyll metabolic pathway [51,52]. Many transcription factors in the *NAC* and *WRKY* gene families are also involved in the degradation of chlorophyll [53]. However, the regulatory mechanisms that underlie chlorophyll and carotenoid metabolism in colored leaves of ‘Yinbi Shuanghui’ have not been revealed. In this study, transcription factors were extracted from the lavenderblush1 module using 28 chlorophyll and carotenoid metabolism-related genes as decoy genes. A total of five genes highly connected with the lavenderblush1 module were identified as candidate hub genes. The major transcription factor families are *HSF*, *NFY*, *TCP20*, and *WRKY*. To identify putative hub genes, 5 phylogenetic trees were further individually constructed by integration of closely related plant species homologue (Figure S6). In *Ginkgo biloba,* the DEGs encoding TCP family transcription factors showed significantly differential expression in the golden leaf mutant and may influence leaf coloration [19]. In the present study, Unigene0018388 was grouped into pigment metabolism related-TCP clade, implying that it could also play a crucial role in the regulation of pigment synthesis in ‘Yinbi Shuanghui’. These results indicate that the regulatory mechanisms in colored leaves involve complex networks.

### 4.3. The Expression of Genes Associated with Carotenoid Metabolism in Colored Leaves impedes the Synthesis of Carotenoids

Carotenoids are found mainly in plant chloroplasts and many flowers and fruits. They also play important roles in plant photosynthesis. The precursor of carotenoid biosynthesis is isoprene pyrophosphate (IPP), which is synthesized by the 2-C-methyl-D-erythritol-4-phosphate (MEP) pathway in the plastid. DXS plays a key role in the regulation of terpenoid synthesis and is the first and rate-limiting enzyme in the MEP pathway. The enzyme is localized in the thylakoids. Excessive expression of *DXS2* can effectively promote the accumulation of carotenoids and isoprene [54]. A significant increase in chlorophyll and carotene in leaves of *A. thaliana* overexpressing the *StDXS*1 gene [55]. Carotenoids and chlorophyll and other endogenous hormones (such as ABA) in *Daucus carota* and *A. thaliana* transformed with the *DXS* are increased [56]. The synthesis of carotenoids starts with geranylgeranyl diphosphate (GGPP) and produces colorless phytoene under the action of PSY. PSY is a key regulatory enzyme that determines the total amount of carotenoid accumulation in plants [57]. The relative expression of *PSY* in *Capsicum annuum* and *Actinidia macrosperma* was high and the carotenoid content was also increased [58,59]. In transgenic plants of *Lycopersicon esculentum*, overexpression of *PSY* resulted in a significant increase in carotenoid content in the seed coat, cotyledon, and hypocotyl [60]. In this study, the genes encoding DXS and PSY in colored leaves showed significant downregulation during the three stages of leaf development, but *PSY* expression was seen gradually to increase. qRT-PCR further confirmed the presence of the *DXS* and *PSY*. Their expression in colored leaves was lower than that in green leaves throughout development. In addition, the *PSY* was enriched in the lavenderblush1 module in WGCNA, indicating that *PSY* affects the carotenoid synthesis and causes a decrease in carotenoid accumulation.

Carotenoid degradation also affects the carotenoid content. There are two main carotenoid cleavage pathways in plants, which involved carotenoid dioxygenase (CCD) and NCED [61]. NCEDs are rate-limiting enzymes that control the conversion of carotenoids to ABA by catalyzing the formation of the ABA precursor xanthophyll [62]. Nine *NCED* have been identified in *A. thaliana*, of which five are in plastids. The regulation of *AtNCED*3 can affect the carotenoid content of the plastid membrane [63]. For example, during the process of peony leaf color change, the *NCED* was upregulated, resulting in a decrease in carotenoid accumulation [64]. Our study found that the gene encoding NCED was upregulated in colored leaves and the carotenoid content in these leaves was significantly lower than that in green leaves during the mature leaf stages. The results are consistent with those found in other leaf color mutants. We conclude that the upregulated expression of *NCED* promotes the degradation of carotenoids, resulting in a decrease in carotenoid content in the colored leaves.

## 5. Conclusions

In our study, the physiological and transcriptome sequences of colored and green leaves of *O. fragrans* ‘Yinbi Shuanghui’ were studied at different developmental stages. Low chlorophyll content and abnormal chloroplast structure were observed in colored leaves. Transcriptome data identified DEGs and pathways related to chlorophyll and carotenoid metabolism. In addition, the association between the co-expressed gene modules formed by DEGs and the physiological indicators was analyzed by WGCNA. Five hub genes involved in regulating chlorophyll and carotenoid metabolism were identified. Consequently, the gene expression related to chlorophyll degradation, carotenoid synthesis, and *GLK* may be related to the formation of colored leaves. These genes would be taken as the candidate genes for our future transgenic improvement. Our research will supply a valuable gene resource for guided plant breeding.

## Figures and Tables

**Table 1:** Comparison of pigment content in colored and green leaf.

Sample	Chlorophyll (mg·g^−1^)	Chlorophyll a (mg·g^−1^)	Chlorophyll b (mg·g^−1^)	Carotenoid (mg·g^−1^)	Chl a/Chl b
GA	0.89 ± 0.02d	0.41 ± 0.00d	0.47 ± 0.02d	0.19 ± 0.00d	0.88 ± 0.03d
GB	2.13 ± 0.07b	1.21 ± 0.07b	0.92 ± 0.03b	0.40 ± 0.03b	1.31 ± 0.08b
GC	2.97 ± 0.01a	1.88 ± 0.03a	1.09 ± 0.03a	0.65 ± 0.00a	1.73 ± 0.07a
CA	1.00 ± 0.01d	0.50 ± 0.01d	0.50 ± 0.02d	0.23 ± 0.01d	1.01 ± 0.05c
CB	1.58 ± 0.08c	0.88 ± 0.05c	0.70 ± 0.03c	0.31 ± 0.02c	1.26 ± 0.02bc
CC	2.14 ± 0.05b	1.23 ± 0.01b	0.91 ± 0.05b	0.32 ± 0.00c	1.35 ± 0.07b

The colored leaf in young leaf stage (CA), leaf-expanding stage (CB), and mature leaf stage (CC); the green leaf in young leaf stage (GA), leaf-expanding stage (GB), and mature leaf stage (GC); Different letters denote significant differences according to Tukey test at the 0.05 level.

**Table 2:** Summary of sequencing data.

Sample	Raw Read	Clean Read	GC%	Base Pair	Q20 (%)	Q30 (%)
CA-1	50136154	48697992 (97.13%)	45.75%	7123264976	6965309247 (97.78%)	6650946216 (93.37%)
CA-2	47247834	45806568 (96.95%)	47.08%	6692400673	6537141040 (97.68%)	6229471306 (93.08%)
CA-3	43851748	42713330 (97.4%)	46.62%	6255173639	6122879028 (97.89%)	5853739955 (93.58%)
CB-1	41291246	40007934 (96.89%)	46.41%	5851697788	5719672342 (97.74%)	5456822949 (93.25%)
CB-2	39691580	38508004 (97.02%)	46.49%	5630898128	5499410453 (97.66%)	5240305903 (93.06%)
CB-3	41987310	40869684 (97.34%)	46.78%	5982309095	5851983440 (97.82%)	5589280458 (93.43%)
CC-1	40240238	38318272 (95.22%)	47.11%	5496433178	5292402611 (96.29%)	4930302996 (89.70%)
CC-2	47464442	45485024 (95.83%)	46.30%	6547342914	6313888832 (96.43%)	5896493760 (90.06%)
CC-3	45207378	43368058 (95.93%)	46.12%	6246637041	6025421211 (96.46%)	5629644889 (90.12%)
GA-1	47923416	46863062 (97.79%)	46.51%	6920028257	6813658441 (98.46%)	6592998268 (95.27%)
GA-2	52077614	49301610 (94.67%)	47.43%	7040828776	6764478452 (96.08%)	6283883279 (89.25%)
GA-3	47559986	45334370 (95.32%)	47.04%	6503246887	6258755974 (96.24%)	5827855536 (89.61%)
GB-1	47687778	45908678 (96.27%)	46.64%	6616481347	6389425806 (96.57%)	5978986991 (90.37%)
GB-2	40856522	38407958 (94.01%)	46.54%	5441768736	5187774775 (95.33%)	4761428460 (87.50%)
GB-3	50998640	49742274 (97.54%)	46.50%	7337535698	7216493845 (98.35%)	6971524292 (95.01%)
GC-1	45633624	44576264 (97.68%)	46.68%	6583222002	6478966906 (98.42%)	6263761501 (95.15%)
GC-2	52890664	51635718 (97.63%)	47.04%	7626415754	7502551431 (98.38%)	7248121801 (95.04%)
GC-3	55658706	54186622 (97.36%)	46.75%	7995143055	7859478736 (98.30%)	7585090670 (94.87%)

The colored leaf in young leaf stage (CA), leaf-expanding stage (CB), and mature leaf stage (CC); the green leaf in young leaf stage (GA), leaf-expanding stage (GB), and mature leaf stage (GC);1-3: Three biological replicates of each leaf sample; Raw reads: original number of reads obtained by sequencing; Clean reads: number of reads after removing low-quality reads and trimming adapter sequences; GC%: percentage of G and C in total bases; Base pair: number of DNA base pair; Q20: Phred score, indicates 99% accuracy of sequenced bases; Q30: Phred score, indicates 99.9% accuracy of sequenced bases.

**Table 3:** High quality clean reads mapped to the reference sequence.

Sample	All Reads Num	UniqueMapped Reads	Multiple Mapped Reads	Mapping Ratio
CA-1	47837392	35574913	5619933	86.11%
CA-2	45460760	34139028	5403742	86.98%
CA-3	41853268	31431109	4984757	87.01%
CB-1	39921676	30448781	4373979	87.23%
CB-2	38064446	29044117	4312621	87.63%
CB-3	40736136	31124068	4696715	87.93%
CC-1	38049842	29707713	3795059	88.05%
CC-2	45215906	35061304	4497542	87.49%
CC-3	43259684	33546908	4150829	87.14%
GA-1	46725254	35483751	4884461	86.39%
GA-2	48854482	36860201	5709217	87.14%
GA-3	45201006	34009232	5270798	86.90%
GB-1	45666432	34822812	5256176	87.76%
GB-2	38305776	28947778	4418419	87.10%
GB-3	49619384	37946864	5267936	87.09%
GC-1	44405898	35133689	4016541	88.16%
GC-2	51159608	40525142	4731774	88.46%
GC-3	53974230	42462991	4936445	87.82%

The colored leaf in young leaf stage (CA), leaf-expanding stage (CB), and mature leaf stage (CC); the green leaf in young leaf stage (GA), leaf-expanding stage (GB), and mature leaf stage (GC);1-3: Three biological replicates of each leaf sample; All Reads Num: total number of reads after ribosome removal; Unique Mapped Reads: number of reads on unique alignment with reference sequence; Multiple Mapped Reads: number of reads on multiple alignments with reference sequence; Mapping Ratio = (Unique Mapped Reads + Multiple Mapped Reads)/All Reads Num.

**Table 4:** Distribution of genes associated with leaf color formation in modules.

Pathway	Chlorophyll metabolism	Carotenoid biosynthesis
all(7005)	71	40
blueviolet(248)	1	2
brown4(236)	2	2
coral(1289)	20	1
darkolivegreen2(1404)	9	6
darkolivegreen4(645)	1	3
honeydew1(274)	5	1
lavenderblush1(1466)	14	14
lightslateblue(7)	0	0
mediumpurple2(682)	6	8
navajowhite(8)	0	0
orange(457)	6	2
plum(42)	0	0
plum1(136)	3	0
sienna4(111)	4	1

**Table 5:** Candidate hub genes for regulation of chlorophyll metabolism and carotenoid metabolism in the lavenderblush1 modules.

Gene Name	Descriptions
Unigene0018388	transcription factor TCP20 isoform X2(TCP20)
Unigene0027439	WRKY transcription factor 3(WRKY3)
Unigene0036235	nuclear transcription factor Y subunit C-9-like isoform X1(NFYC9)
Unigene0000179	probable WRKY transcription factor 3 isoform X2(WRKY4)
Unigene0029411	heat stress transcription factor A-7a-like (HSFA2)

## Supplementary Materials

The following are available online at https://www.mdpi.com/2218-273X/10/4/549/s1, Table S1: Primer sequences used for qRT-PCR, Table S2: Top 20 candidate genes among the annotated DEGs of colored and green leaf in different stages, Table S3: Metabolic pathways in which DEGs are significantly enriched in CA vs. GA, Table S4: Metabolic pathways in which DEGs are significantly enriched in CB vs. GB, Table S5: Metabolic pathways in which DEGs are significantly enriched in CC vs. GC, Table S6: Annotation of transcription factors in lavenderblush1 module, Table S7: 28 chlorophyll metabolism- and carotenoid metabolism-related genes in the lavenderblush1 module, Figure S1: PCA of all samples, Figure S2: Volcano plots of differential gene expression of all samples in different leaf stages, Figure S3: Module hierarchical clustering tree, Figure S4: Classification of transcription factors in the lavenderblush1 module, Figure S5: The cluster of different bioreps in Figure 7; Figure S6: Phylogenetic tree derived from amino acid sequences of 5 hub genes.
