# Supplementary material for: Biochemical and Comparative Transcriptome Analyses Reveal Key Genes Involved in Major Metabolic Regulation Related to Colored Leaf Formation in Osmanthus fragrans ‘Yinbi Shuanghui’ during Development
Source: Biomolecules. 2020 Apr 4;10(4):549. doi: 10.3390/biom10040549 (PMC7226453; doi:10.3390/biom10040549)
Supplement: Supplementary file 1 [file biomolecules-10-00549-s001.pdf]

biomolecules

MDPI

Article

Biochemical and Comparative Transcriptome Analyses Reveal Key Genes Involved in Major Metabolic Regulation Related to Colored Leaf Formation in *Osmanthus fragrans* 'Yinbi Shuanghui' during Development

Xuan Chen [1,3], Xiulian Yang [1,2], Jun Xie [3], Wenjie Ding [1,2], Yuli Li [1,2], Yuanzheng Yue [1,2,*] and Lianggui Wang [1,2,*]

1 Key Laboratory of Landscape Architecture, Jiangsu Province, College of Landscape Architecture, Nanjing Forestry University, No. 159 Longpan Road, Nanjing 210037, China; chenxuan@njts.edu.cn (X.C.); xly@njfu.edu.cn (X.Y.); wenjieding@njfu.edu.cn (W.D.); chestnutlyl@njfu.edu.cn (Y.L.)

2 Co-Innovation Center for Sustainable Forestry in Southern China, Nanjing Forestry University, Nanjing 210037, China

3 College of Fine Arts, Nanjing Normal University of Special Education, No.1 Shennong Road, Nanjing 210038, China; xiejun@njts.edu.cn

* Correspondence: yueyuanzheng@njfu.edu.cn (Y.Y.); wlg@njfu.com.cn (L.W.); Tel.: +86-138-0900-7625 (L.W.)

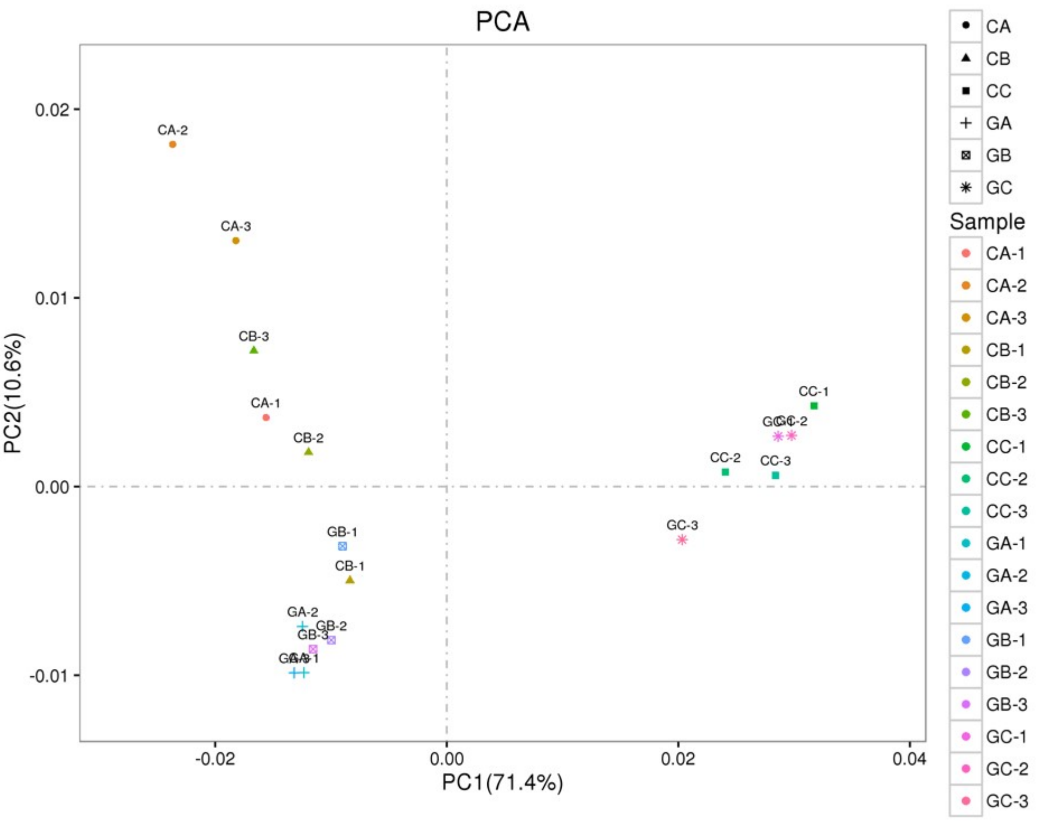

Figure S1. PCA of all samples.

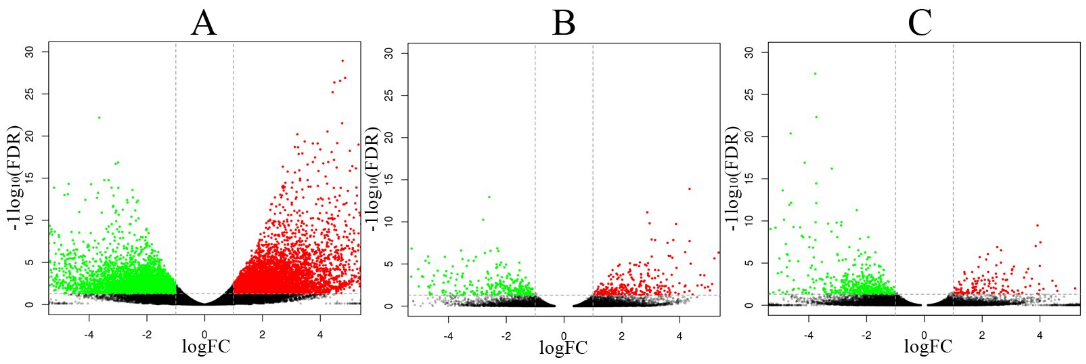

Figure S2. Volcano plots of differential gene expression of all samples in different leaf stages. (A) CA-VS-GA; (B) CB-VS-GB; (C) CC-VS-GC. The colored leaf in young leaf stage(CA), leaf-expanding stage(CB), and mature leaf stage(CC); the green leaf in young leaf stage(GA), leaf-expanding stage(GB), and mature leaf stage(GC)**.** The abscissa represents the logarithm of the multiple of the difference between the two samples, and the ordinate represents the negative Log10 value of the FDR of the two samples. Red (up-regulated expression) and green (down-regulated expression) dots indicate that there is a difference in the expression level of the gene (judgment criterion is FDR <0.05, more than a multiple of difference), and black dots are no difference.

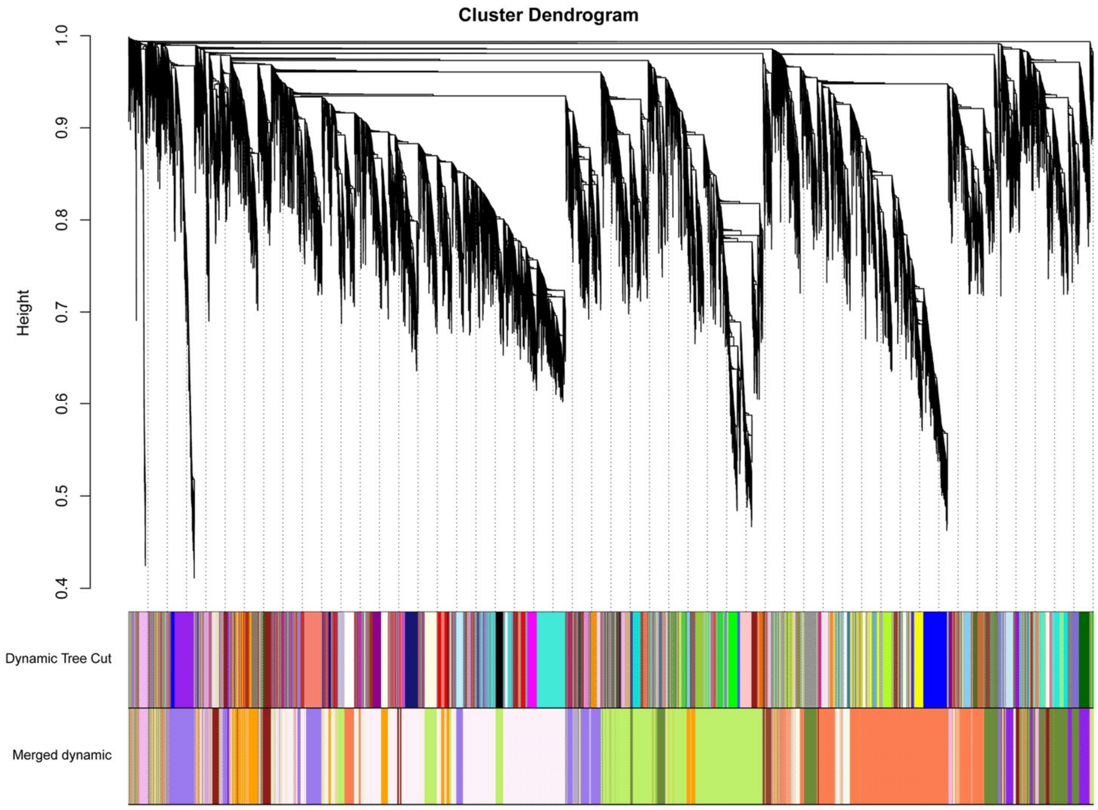

Figure S3. Module hierarchical clustering tree. **(A)** Gene clustering tree based on correlation between gene expression levels; **(B)** Modules based on clustering results; **(C)** Module partitioning based on module similarity for modules with similar expression patterns.

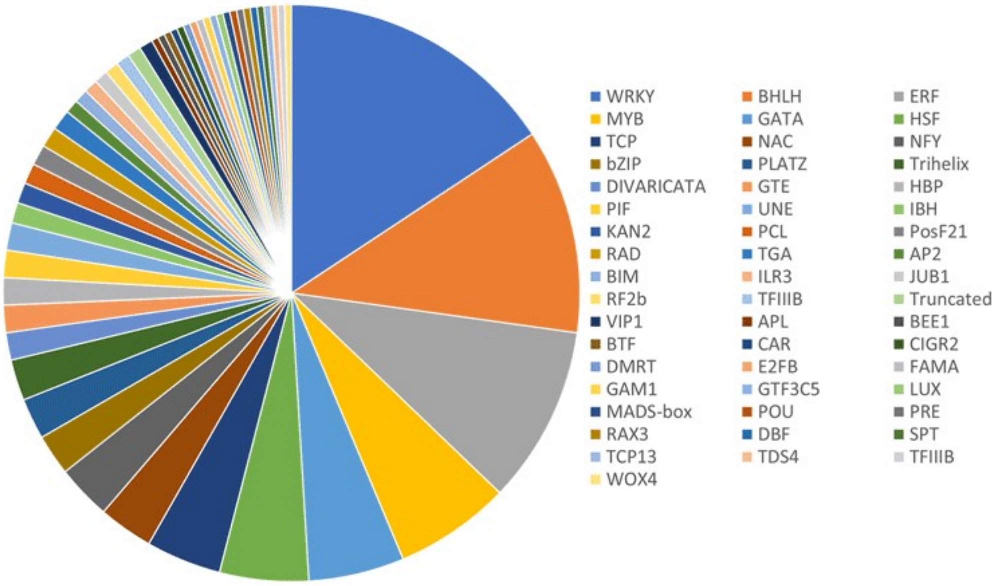

Figure S4. Classification of transcription factors in the lavenderblush1 module

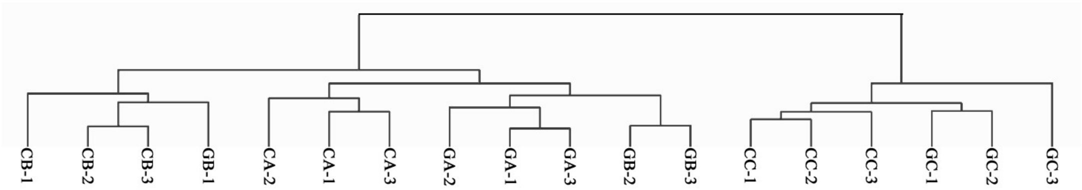

Figure S5. The cluster of different bioreps in Figure 7.

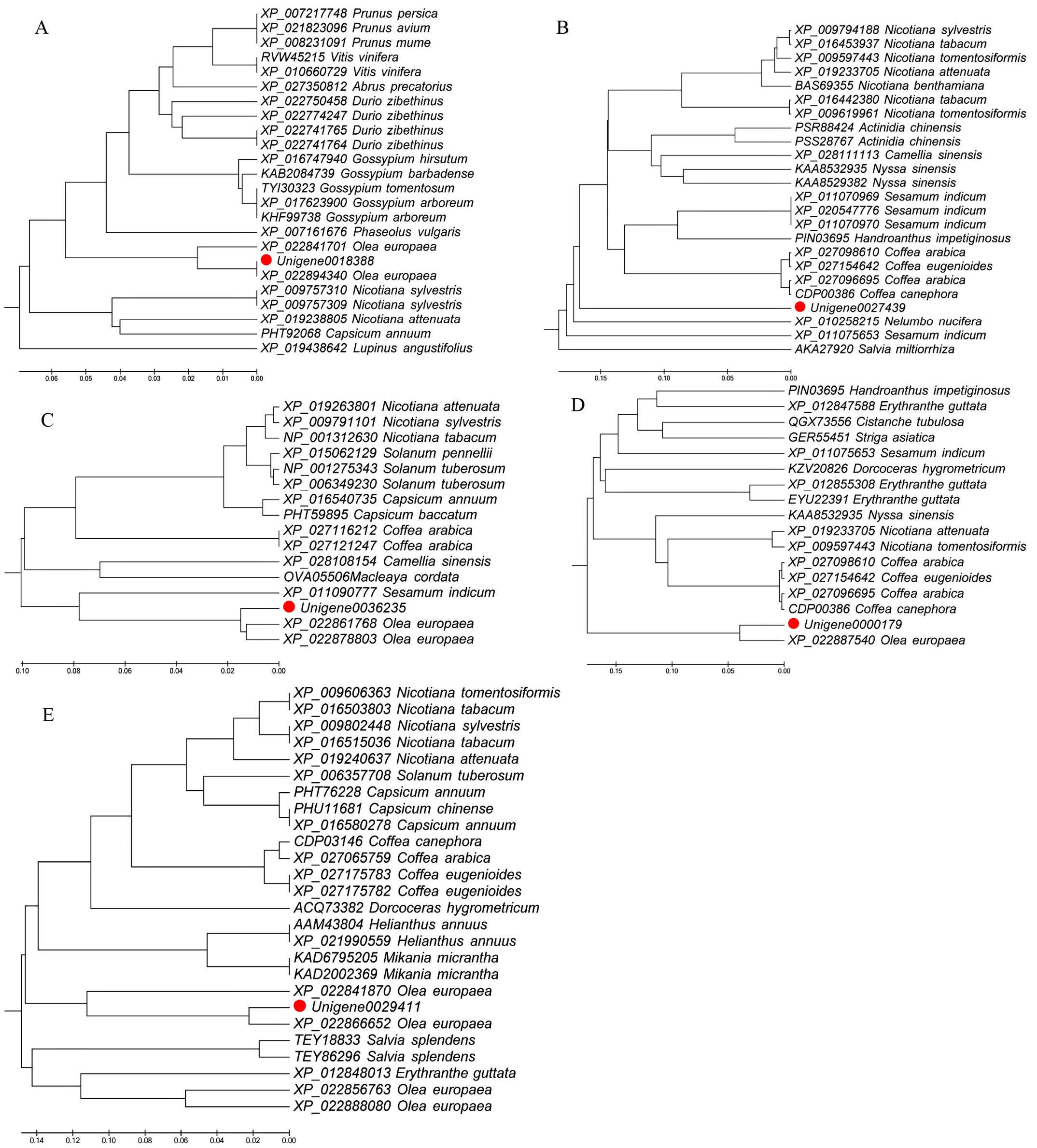

Figure S6. Phylogenetic tree derived from amino acid sequences of 5 hub genes. (**A**)Unigene0018388, TCP20; (**B**)Unigene0027439, WRKY3; (**C**)Unigene0036235, NFYC9; (**D**) Unigene0000179, WRKY4; (**E**)Unigene0029411, HSFA2; Five hub genes isolated in this study was with red circle.

Table S1. Primer sequences used for qRT-PCR.

Gene name	Primer sequence (5′-3′)
Unigene0032350-F	TTGTGGGAGATGGGTGCTTTG
Unigene0032350-R	TGGCAACAGGGACAATGGAC
Unigene0050901-F	TCTGCTGCCTCTGGCGTAT
Unigene0050901-R	CTCCTTCTAATGGGGCCTACAC
Unigene0011700-F	TGCCCAAATCCTCCAAATCA
Unigene0011700-R	CCATGTCAAACCATCTTCCCTAA
Unigene0030710-F	ATCGCCATTGATAGGAACCG
Unigene0030710-R	TCACCAACCTCGCATACCC
Unigene0039679-F	GACCTGCGAGATTTCCGAGTA
Unigene0039679-R	GCAGCATTTGTCCAGAGGGTA
Unigene0003650-F	GTTGGCTACTCCGATTCCTTG
Unigene0003650-R	TTACGCTTGCCGATTTACAGA
Unigene0031455-F	GGCAAGCATCATCTCATTCACA
Unigene0031455-R	GGGCAGGCATTTACTCCACT
Unigene0036601-F	TGAAGTGGATAGGCTTGAGTGC
Unigene0036601-R	CCGAGAAATGGACCAGGATG
Unigene0044025-F	TGGCAGTTTCTGGACTTGTGATT
Unigene0044025-R	ATCTGGCTTCCAACCATCTTTTT
Unigene0054034-F	GAAGGTGGTTCTTTCGTAGCC
Unigene0054034-R	ATTGGCAGAGCACTTGACGAG
Unigene0018384-F	AGTAAGGCTGCTAAGGAGTGGG
Unigene0018384-R	ACCATACCCTTGAACATTACCG
Unigene0020260-F	ACCCAACAACCCGATGAGACA
Unigene0020260-R	CAAGTGCGTGCTTTCCAACAT
Unigene0026778-F	CCCCGAGATTGACCACGAA
Unigene0026778-R	ACTTGGGCTAAAGCGGCAC
Unigene0029232-F	TCTCGTCACATTCCTCGTCATC
Unigene0029232-R	TTCCTCCTGTTTGAACTCTTGCT
Unigene0044911-F	GATTTCTTCGCCATAGCCG
Unigene0044911-R	CGTGCCTAGTTTACACCCTCC
Unigene0044913-F	GTTCACCCTTAGCCCTGTTTTC
Unigene0044913-R	TTCCCAACTTGAGGTCCTAACA

Table S2. Top 20 candidate genes among the annotated DEGs of colored and green leaf in different stages

GeneID	log2Ratio(G/C)	FDR	Description
**CA-VS-GA**			
Unigene0020511	-13.38	5.60×10^{-14}	RS30, ribosomal protein 30 40S small ribosomal subunit [*Thalassiosira pseudonana*]
Unigene0010908	12.64	1.57×10^{-14}	raucaffricine-O-beta-D-glucosidase-like [*Sesamum indicum*]
Unigene0017434	-12.39	5.00×10^{-12}	60S ribosomal protein L23 [*Capsicum annuum*]
Unigene0019443	-12.27	2.11×10^{-10}	ribosomal protein S29 subunit [*Chlamydomonas* sp. ICE-L]
Unigene0001381	-12.03	9.52×10^{-07}	40S ribosomal protein S28-B [*Pyrus* x *bretschneideri*]
Unigene0037023	-11.99	7.01×10^{-13}	RL11A, ribosomal protein 11A 60S large ribosomal subunit [*Thalassiosira pseudonana*]
Unigene0033316	-11.99	5.95×10^{-11}	thiamine biosynthetic enzyme [*Monoraphidium neglectum*]
Unigene0009240	-11.88	6.04×10^{-06}	ATP synthase subunit 9, mitochondrial, partial [*Jatropha curcas*]
Unigene0002868	-11.83	7.39×10^{-11}	probable mannitol dehydrogenase [*Gossypium arboreum*]
Unigene0005002	-11.75	4.04×10^{-10}	KOW-like protein [*Cynara cardunculus*]
Unigene0014347	-11.61	4.48×10^{-06}	Ribosomal protein S5 family protein isoform 1 [*Theobroma cacao*]
Unigene0004133	-11.59	2.73×10^{-09}	40S ribosomal protein S13-like [*Populus euphratica*]
Unigene0012304	-11.57	7.34×10^{-08}	60S ribosomal protein L14, mitochondrial-like [*Beta vulgaris* subsp. vulgaris]
Unigene0015550	-11.54	5.71×10^{-06}	thiazole biosynthetic enzyme, partial [*Guillardia theta*]
Unigene0055782	-11.54	3.37×10^{-11}	2,3-dihydro-2,3-dihydroxybenzoate dehydrogenase [*Cyanidioschyzon merolae*]
Unigene0023449	-11.49	6.36×10^{-08}	60s ribosomal protein l18a [*Nannochloropsis gaditana*]
Unigene0054244	-11.25	7.18×10^{-09}	50S ribosomal protein L13, partial [*Glycine soja*]
Unigene0063753	-11.21	3.28×10^{-05}	F-type H+-transporting ATPase subunit delta [*Galdieria sulphuraria*]
Unigene0052586	-11.16	6.91×10^{-19}	cytochrome c oxidase subunit I, partial (mitochondrion) [*Lingulodinium polyedrum*]
Unigene0059644	-11.15	1.06×10^{-10}	cytochrome c oxidase subunit 2 (mitochondrion) [*Nephroselmis olivacea*]
**CB-VS-GB**			
Unigene0055377	-11.74	4.89×10^{-09}	30S ribosomal protein S4, chloroplastic-like [*Pyrus* x *bretschneideri*]
Unigene0012304	-11.58	1.39×10^{-07}	60S ribosomal protein L14, mitochondrial-like [*Beta vulgaris* subsp. vulgaris]
Unigene0057000	-11.29	8.82×10^{-09}	30S ribosomal protein S10, chloroplastic-like [*Pyrus* x *bretschneideri*]
Unigene0052586	-11.21	1.36×10^{-23}	cytochrome c oxidase subunit I, partial (mitochondrion) [*Lingulodinium polyedrum*]
Unigene0060614	-11.03	5.67×10^{-13}	cytochrome c oxidase subunit 3 (mitochondrion) [*Chlorella* sp. ArM0029B]
Unigene0059644	-11.02	1.26×10^{-09}	cytochrome c oxidase subunit 2 (mitochondrion) [*Nephroselmis olivacea*]

Unigene0056859	-11.01	1.61×10^{-04}	NAD(P) transhydrogenase subunit alpha [*Monoraphidium neglectum*]
Unigene0054244	-10.89	7.44×10^{-07}	50S ribosomal protein L13, partial [*Glycine soja*]
Unigene0060874	-10.71	7.22×10^{-08}	glyceraldehyde-3-phosphate dehydrogenase [*Marchantia polymorpha*]
Unigene0055678	-10.55	7.73×10^{-05}	superoxide dismutase [*Chlamydomonas reinhardtii*]
Unigene0060253	-10.39	1.50×10^{-03}	cobalamin-independent methionine synthase [*Coccomyxa subellipsoidea*]
Unigene0054589	-10.29	1.80×10^{-03}	30S ribosomal protein S2 [*Aegilops tauschii*]
Unigene0012305	-10.28	1.80×10^{-04}	50S ribosomal protein L5, organellar chromatophore-like [*Pyrus* x *bretschneideri*]
Unigene0061856	-10.26	8.01×10^{-07}	vitamine B12-independent methionine synthase [*Cyanidioschyzon merolae*]
Unigene0016983	-10.24	1.67×10^{-03}	vitamine B12-independent methionine synthase [*Cyanidioschyzon merolae*]
Unigene0053233	-10.23	7.40×10^{-03}	3-oxoacyl-[acyl-carrier-protein] reductase, partial [*Cajanus cajan*]
Unigene0013691	-10.22	2.62×10^{-03}	probable trehalose-phosphate phosphatase J [*Sesamum indicum*]
Unigene0054851	-10.20	1.61×10^{-04}	vitamine B12-independent methionine synthase [*Cyanidioschyzon merolae*]
Unigene0056578	-10.20	3.13×10^{-03}	ribosomal protein L12 [*Kryptoperidinium foliaceum*]
Unigene0059151	-10.06	6.22×10^{-04}	glyceraldehyde-3-phosphate dehydrogenase C2 [*Arachis hypogaea]*
**CC-VS-GC**			
Unigene0054715	-12.21	8.94×10^{-03}	crystal protein [*Nannochloropsis gaditana*]
Unigene0064698	-12.12	4.46×10^{-04}	camp-regulated d2 protein [*Chrysochromulina* sp. CCMP291]
Unigene0014134	11.51	9.30×10^{-12}	cytochrome c oxidase subunit 3 [*Chlorella* sp. ArM0029B]
Unigene0006358	10.71	4.07×10^{-11}	Phosphoserine phosphatase [*Ectocarpus siliculosus*]
Unigene0058494	10.60	1.15×10^{-04}	fructose-1,6-bisphosphatase [*Phaeodactylum tricornutum*]
Unigene0000072	-10.59	1.07×10^{-08}	ribulose bisphosphate carboxylase small chain 1A [*Arabidopsis thaliana*]
Unigene0056333	-10.40	7.87×10^{-04}	60S ribosomal protein L27-3 [*Arabidopsis thaliana*]
Unigene0056548	-10.38	1.02×10^{-05}	fructose-bisphosphate aldolase [*Arabidopsis thaliana*]
Unigene0055152	-10.35	4.32×10^{-06}	PYK10 [*Arabidopsis thaliana*]
Unigene0062828	10.21	3.26×10^{-04}	fructose-bisphosphate aldolase cytoplasmic isozyme [*Gossypium hirsutum*]
Unigene0066133	10.20	3.59×10^{-03}	cytochrome c oxidase subunit I, partial (mitochondrion) [*Lingulodinium polyedrum*]
Unigene0053528	10.19	6.90×10^{-04}	60S acidic ribosomal protein P2B-like [*Ziziphus jujuba*]
Unigene0058280	10.16	1.09×10^{-04}	cytochrome oxidase subunit I, partial (mitochondrion) [*Cyclotella* sp. MBTD-CMFRI-S052]
Unigene0057226	-10.15	5.43×10^{-03}	ORF40m [*Pinus koraiensis*]
Unigene0053978	10.13	5.21×10^{-04}	glyceraldehyde-3-phosphate dehydrogenase, partial [*Sphagnum cuspidatum*]

Unigene0054433	-10.08	3.23×10^{-03}	glyceradehyde-3-phosphate dehydrogenase, partial [*Brassica rapa* subsp. chinensis]
Unigene0058969	-10.08	6.90×10^{-04}	adenosylhomocysteinase, partial [*Arabidopsis thaliana*]
Unigene0059680	-9.99	6.84×10^{-04}	peroxidase prxr1 [*Arabidopsis thaliana*]
Unigene0055021	-9.95	5.02×10^{-04}	PYK10 [*Arabidopsis thaliana*]
Unigene0061140	9.91	8.01×10^{-06}	fructose-bisphosphate aldolase [*Medicago truncatula*]

Table S3. Metabolic pathways in which DEGs are significantly enriched in CA VS GA

Pathway	Candidate genes with pathway annotation	P-value	Q-value
**_up Pathway Enrichment**			
DNA replication	41 (3.95%)	0	0
Anthocyanin biosynthesis	13 (1.25%)	0.000001	0.000088
Ribosome biogenesis in eukaryotes	51 (4.92%)	0.000005	0.000189
Homologous recombination	28 (2.7%)	0.000006	0.000189
Pyrimidine metabolism	53 (5.11%)	0.000008	0.000198
RNA transport	69 (6.65%)	0.000031	0.000634
Isoquinoline alkaloid biosynthesis	16 (1.54%)	0.000041	0.0007
RNA degradation	54 (5.21%)	0.000151	0.00229
Nitrogen metabolism	17 (1.64%)	0.000195	0.002623
Tyrosine metabolism	20 (1.93%)	0.000405	0.004884
Flavonoid biosynthesis	13 (1.25%)	0.00047	0.004884
Base excision repair	20 (1.93%)	0.000484	0.004884
ABC transporters	23 (2.22%)	0.000711	0.00623
Purine metabolism	56 (5.4%)	0.000721	0.00623
Nucleotide excision repair	27 (2.6%)	0.002022	0.015659
Mismatch repair	19 (1.83%)	0.002071	0.015659
**_down Pathway Enrichment**			
Ribosome	127 (14.46%)	0	0
Photosynthesis	24 (2.73%)	0.000189	0.01087
Oxidative phosphorylation	49 (5.58%)	0.00044	0.016872
RNA polymerase	18 (2.05%)	0.00104	0.02991
Protein processing in endoplasmic reticulum	63 (7.18%)	0.001546	0.035556

Table S4. Metabolic pathways in which DEGs are significantly enriched in CB VS GB

Pathway	Candidate genes with pathway annotation	P-value	Q-value
**_up Pathway Enrichment**			
Protein processing in endoplasmic reticulum	35 (47.3%)	0	0
Flavonoid biosynthesis	5 (6.76%)	0.00002	0.000411
Monoterpenoid biosynthesis	3 (4.05%)	0.000062	0.000862
Anthocyanin biosynthesis	3 (4.05%)	0.001214	0.012749
**_down Pathway Enrichment**			
Starch and sucrose metabolism	12 (16.44%)	0.000049	0.001817
Selenocompound metabolism	5 (6.85%)	0.000071	0.001817
Ribosome	15 (20.55%)	0.000406	0.006902
Tryptophan metabolism	4 (5.48%)	0.00093	0.011863
Phenylpropanoid biosynthesis	7 (9.59%)	0.001827	0.018632

Table S5. Metabolic pathways in which DEGs are significantly enriched in CC VS GC

Pathway	Candidate genes with pathway annotation	P-value	Q-value
**_up Pathway Enrichment**			
Biosynthesis of amino acids	9 (20.93%)	0.000167	0.00619

Table S6. Annotation of transcription factors in lavenderblush1 module

GeneID	Description
Unigene0000179	probable WRKY transcription factor 3 isoform X2
Unigene0000410	probable WRKY transcription factor 41
Unigene0001933	WRKY family transcription factor
Unigene0001980	GATA transcription factor 26-like
Unigene0002385	transcription factor E2FB
Unigene0002573	transcription factor RF2b-like
Unigene0003774	MYB-related transcription factor
Unigene0004528	GATA transcription factor 21-like
Unigene0005430	transcription factor GTE12
Unigene0005874	transcription factor bHLH104
Unigene0006009	probable transcription factor KAN4 isoform X2
Unigene0006291	probable WRKY transcription factor 70
Unigene0006390	NAC transcription factor 29
Unigene0006405	transcription factor PCL1-like
Unigene0006876	heat stress transcription factor A-3-like
Unigene0007067	transcription factor bHLH112 isoform X1
Unigene0007170	ethylene-responsive transcription factor RAP2-4
Unigene0007983	GATA transcription factor 7-like
Unigene0008228	transcription factor bHLH51
Unigene0008469	transcription factor GAMYB-like isoform X2
Unigene0008882	transcription factor PIF1-like
Unigene0009467	PLATZ transcription factor family protein
Unigene0009514	transcription factor BIM2-like
Unigene0009995	transcription factor DIVARICATA-like
Unigene0010072	PLATZ transcription factor family protein isoform 1
Unigene0011229	transcription factor VIP1 isoform X2
Unigene0011508	transcription factor bHLH112 isoform X1
Unigene0011524	PLATZ transcription factor family protein isoform 1
Unigene0011657	nuclear transcription factor Y subunit A-10-like isoform X2
Unigene0011936	transcription factor bHLH128-like isoform X1
Unigene0014551	bZIP transcription factor 60-like
Unigene0014722	probable WRKY transcription factor 31
Unigene0014903	ethylene-responsive transcription factor ERF107-like
Unigene0014940	WRKY transcription factor 2
Unigene0014941	WRKY transcription factor 2
Unigene0015025	transcription factor RAX3-like
Unigene0015292	ethylene-responsive transcription factor RAP2-10-like
Unigene0015293	ethylene-responsive transcription factor ERF011
Unigene0015457	ethylene-responsive transcription factor RAP2-10-like
Unigene0015842	transcription factor MYB24-like
Unigene0015895	transcription factor TCP9
Unigene0015896	transcription factor TCP9
Unigene0015958	nuclear transcription factor Y subunit C-3-like isoform X1
Unigene0016235	WRKY transcription factor 85
Unigene0016430	nuclear transcription factor Y subunit A-1-like

Unigene0016677	PLATZ transcription factor family protein isoform 1
Unigene0016692	transcription factor TFIIIB component B'' homolog isoform X5
Unigene0016695	transcription factor TFIIIB component B'' homolog isoform X5
Unigene0017315	bZIP transcription factor
Unigene0017599	transcription factor 25 isoform X2
Unigene0017603	NAC transcription factor 25-like
Unigene0017604	NAC transcription factor 56
Unigene0017625	ethylene-responsive transcription factor 3-like
Unigene0017647	WRKY transcription factor 2
Unigene0017649	WRKY transcription factor 2
Unigene0017799	WRKY transcription factor 85
Unigene0017967	probable WRKY transcription factor 70
Unigene0018213	transcription factor TCP9
Unigene0018279	trihelix transcription factor ASIL2
Unigene0018320	ethylene-responsive transcription factor ERF107-like
Unigene0018321	ethylene-responsive transcription factor ERF107-like
Unigene0018388	transcription factor TCP20 isoform X2
Unigene0018389	transcription factor TCP20 isoform X2
Unigene0018455	transcription factor EGL1-like
Unigene0018484	transcription factor bHLH30
Unigene0018486	transcription factor bHLH30-like
Unigene0018588	transcription factor bHLH18-like
Unigene0018742	nuclear transcription factor Y subunit B-8-like
Unigene0019270	transcription factor MYB1R1 isoform X2
Unigene0019424	ethylene-responsive transcription factor ERF023-like
Unigene0019522	transcription factor MYB59
Unigene0019523	MYB transcription factor MYB145, partial
Unigene0020473	bHLH transcription factor Upa20
Unigene0020496	myb family transcription factor APL-like isoform X2
Unigene0020507	ethylene-responsive transcription factor RAP2-7 isoform X2
Unigene0021136	transcription factor LUX
Unigene0021390	transcription factor bHLH74-like
Unigene0021646	transcription factor MYB44-like
Unigene0021750	transcription factor ILR3-like isoform X2
Unigene0021830	ethylene-responsive transcription factor SHINE 2
Unigene0021887	ethylene-responsive transcription factor RAP2-12-like
Unigene0022281	NAC transcription factor
Unigene0022315	ethylene-responsive transcription factor ERF113 isoform X1
Unigene0022343	heat stress transcription factor A-3-like
Unigene0022344	heat stress transcription factor A-3 isoform X2
Unigene0022601	transcription factor IBH1-like
Unigene0022602	transcription factor IBH1-like
Unigene0022621	transcription factor MYC4
Unigene0022852	nuclear transcription factor Y subunit A-10-like isoform X1
Unigene0022892	transcription factor IBH1-like
Unigene0022925	GATA transcription factor 24-like
Unigene0023295	heat stress transcription factor A-4c
Unigene0023402	PLATZ transcription factor family protein
Unigene0023403	PLATZ transcription factor family protein
Unigene0023699	ethylene-responsive transcription factor TINY-like

Unigene0023898	basic helix-loop-helix transcription factor
Unigene0024145	trihelix transcription factor ASIL2
Unigene0024382	transcription factor MYB1R1
Unigene0024445	probable WRKY transcription factor 30
Unigene0024560	transcription factor TGA4 isoform X2
Unigene0024913	transcription factor bHLH113 isoform X1
Unigene0024995	transcription factor JUNGBRUNNEN 1-like
Unigene0024996	transcription factor JUNGBRUNNEN 1
Unigene0025046	ethylene-responsive transcription factor ERF010-like
Unigene0025047	ethylene-responsive transcription factor ERF010-like
Unigene0025305	transcription factor bHLH68
Unigene0025384	bZIP transcription factor 53
Unigene0025503	ethylene-responsive transcription factor RAP2-3
Unigene0025773	myb family transcription factor APL-like
Unigene0025993	transcription factor bHLH13-like
Unigene0026006	nuclear transcription factor Y subunit A-1-like
Unigene0026051	transcription factor GTE12
Unigene0026206	probable WRKY transcription factor 70
Unigene0026409	probable WRKY transcription factor 69
Unigene0026582	transcription factor BEE 3-like
Unigene0026806	probable WRKY transcription factor 48
Unigene0026954	transcription factor TCP20
Unigene0026955	transcription factor TCP20
Unigene0026979	heat stress transcription factor A-1-like
Unigene0027151	trihelix transcription factor ASR3 isoform X1
Unigene0027191	ethylene-responsive transcription factor ERF061-like
Unigene0027203	truncated transcription factor CAULIFLOWER A
Unigene0027204	truncated transcription factor CAULIFLOWER A
Unigene0027439	probable WRKY transcription factor 3 isoform X2
Unigene0027440	probable WRKY transcription factor 3 isoform X2
Unigene0027601	transcription factor bHLH18-like
Unigene0027656	probable WRKY transcription factor 50
Unigene0027657	probable WRKY transcription factor 50
Unigene0027701	transcription factor bHLH123
Unigene0027736	heat stress transcription factor B-2a-like
Unigene0028213	transcription factor bHLH149
Unigene0028229	ethylene-responsive transcription factor ERF114, partial
Unigene0028352	transcription factor VIP1-like
Unigene0028671	transcription factor
Unigene0028688	transcription factor bHLH47-like
Unigene0028791	heat stress transcription factor A-1-like
Unigene0028893	heat stress transcription factor B-4-like
Unigene0028905	GATA transcription factor 5-like
Unigene0028906	GATA transcription factor 5-like
Unigene0028992	WRKY transcription factor
Unigene0029115	heat stress transcription factor C-1-like
Unigene0029204	GATA transcription factor 12-like
Unigene0029411	heat stress transcription factor A-7a-like
Unigene0029540	transcription factor TFIIIB component B'' homolog isoform X4
Unigene0029797	transcription factor bHLH48-like isoform X2

Unigene0029923	transcription factor bHLH67 isoform X1
Unigene0029949	MADS-box transcription factor 23
Unigene0030141	transcription factor TCP14-like
Unigene0030150	WRKY transcription factor, partial
Unigene0030173	transcription factor MYB108-like
Unigene0030457	probable WRKY transcription factor 57 isoform X1
Unigene0030459	probable WRKY transcription factor 57 isoform X1
Unigene0030755	probable transcription factor PosF21
Unigene0030812	probable WRKY transcription factor 70
Unigene0030813	probable WRKY transcription factor 70
Unigene0031036	transcription factor FAMA isoform X1
Unigene0031131	transcription factor RADIALIS-like
Unigene0031132	transcription factor RADIALIS-like
Unigene0031133	transcription factor RADIALIS-like
Unigene0031566	transcription factor MYB1R1
Unigene0031612	transcription factor bHLH110
Unigene0031639	scarecrow-like transcription factor PAT1
Unigene0031649	transcription factor DIVARICATA-like
Unigene0031650	transcription factor DIVARICATA-like
Unigene0031855	Doublesex- and mab-3-related transcription factor 3
Unigene0031859	transcription factor PIF3 isoform X2
Unigene0031860	transcription factor PIF3 isoform X2
Unigene0031936	sequence-specific DNA-binding transcription factor
Unigene0032042	transcription factor UNE10
Unigene0032044	transcription factor UNE10
Unigene0032059	ethylene-responsive transcription factor WIN1-like
Unigene0032063	myb family transcription factor APL-like
Unigene0032688	BZIP transcription factor
Unigene0032755	GATA transcription factor 12-like
Unigene0033809	probable WRKY transcription factor 7
Unigene0033928	probable WRKY transcription factor 42
Unigene0034113	trihelix transcription factor GT-2-like
Unigene0034331	BZIP domain class transcription factor
Unigene0034604	WRKY transcription factor
Unigene0035031	transcription factor bHLH137-like
Unigene0035290	probable WRKY transcription factor 69
Unigene0036024	transcription factor GTE7-like
Unigene0036128	transcription factor UNE12
Unigene0036234	nuclear transcription factor Y subunit C-9-like isoform X1
Unigene0036235	nuclear transcription factor Y subunit C-9-like isoform X1
Unigene0036260	probable transcription factor KAN2 isoform X2
Unigene0036261	probable transcription factor KAN2 isoform X2
Unigene0036355	probable transcription factor PosF21
Unigene0036422	ethylene-responsive transcription factor 3-like
Unigene0036844	CAR1 transcription factor
Unigene0037248	transcription factor PCL1-like
Unigene0037586	myb family transcription factor family protein
Unigene0037726	transcription factor BTF3 homolog 4-like
Unigene0037880	transcription factor BIM1
Unigene0038288	transcription factor RF2b-like

Unigene0038741	transcription factor
Unigene0038955	trihelix transcription factor ASIL1-like
Unigene0038979	NAC transcription factor 25
Unigene0039120	GATA transcription factor 28, partial
Unigene0039122	GATA transcription factor 27-like isoform X1
Unigene0039124	GATA transcription factor 26-like
Unigene0039126	GATA transcription factor 26-like
Unigene0039194	ethylene-responsive transcription factor RAP2-4
Unigene0039364	probable WRKY transcription factor 20
Unigene0039366	probable WRKY transcription factor 20
Unigene0039367	probable WRKY transcription factor 20
Unigene0039401	heat stress transcription factor A-8
Unigene0039804	transcription factor bHLH48-like isoform X2
Unigene0040498	transcription factor SPATULA-like
Unigene0040541	transcription factor TGA2
Unigene0040543	transcription factor HBP-1b(c38)-like
Unigene0040544	transcription factor HBP-1b(c38)-like
Unigene0040545	transcription factor TGA2
Unigene0041813	transcription factor UNE12
Unigene0042045	transcription factor bHLH68-like
Unigene0042174	B-zip transcription factor
Unigene0042461	transcription factor PIF7 isoform X1
Unigene0042675	probable WRKY transcription factor 3 isoform X1
Unigene0042783	MYB-related transcription factor
Unigene0043140	general transcription factor 3C polypeptide 5-like
Unigene0043177	transcription factor TCP4-like
Unigene0043178	transcription factor TCP4-like
Unigene0043979	transcription factor APETALA2
Unigene0044650	ethylene-responsive transcription factor RAP2-13
Unigene0044652	ethylene-responsive transcription factor RAP2-4-like
Unigene0044836	AP2-like ethylene responsive transcription factor AIL1 isoform X2
Unigene0044847	transcription factor IIIB 50 kDa subunit
Unigene0044935	transcription factor PCL1-like
Unigene0045268	transcription factor bHLH63
Unigene0045269	transcription factor bHLH63
Unigene0045375	GATA transcription factor 8-like
Unigene0045415	transcription factor DIVARICATA
Unigene0046696	heat stress transcription factor A-5
Unigene0047196	transcription factor HBP-1a-like isoform X2
Unigene0047629	MYB transcription factor
Unigene0047798	WRKY transcription factor 1-like isoform X1
Unigene0048788	probable WRKY transcription factor 71
Unigene0048888	transcription factor HBP-1b(c1)-like isoform X1
Unigene0052711	NAC transcription factor 25-like
Unigene0053711	probable WRKY transcription factor 4
Unigene0054430	transcription factor bHLH112 isoform X2
Unigene0055893	probable WRKY transcription factor 50
Unigene0057266	POU domain, class 3, transcription factor 3-A-like
Unigene0057958	NAC transcription factor 25-like

Unigene0059221	probable WRKY transcription factor 40
Unigene0061833	probable transcription factor PosF21
Unigene0062599	NAC transcription factor 25-like
Unigene0062827	probable WRKY transcription factor 71
Unigene0062839	transcription factor GTE1-like isoform X1
Unigene0063097	ethylene-responsive transcription factor TINY, partial
Unigene0063495	WRKY transcription factor
Unigene0063937	myb family transcription factor family protein
Unigene0064097	transcription factor PRE6-like
Unigene0064312	trihelix transcription factor GTL1-like
Unigene0064348	heat stress transcription factor A-7a-like
Unigene0065673	Ethylene-responsive transcription factor 1B
Unigene0065768	transcription factor ILR3-like
Unigene0065978	basic helix-loop-helix transcription factor
Unigene0066153	transcription factor TCP8
Unigene0066187	GATA transcription factor 21
Unigene0066489	myb family transcription factor APL

Table S7. 28 chlorophyll metabolism- and carotenoid metabolism-related genes in the lavenderblush1 module

GeneID	Connectivity	Symbol
**Chlorophyll metabolism-related genes**		
Unigene0039314	2502.28	NYC1
Unigene0044111	2180.60	HEMH
Unigene0018571	2163.73	CLH1
Unigene0010601	2093.28	CLH2
Unigene0028397	1862.49	HO1
Unigene0011977	1845.96	sll1917
Unigene0029892	1814.30	PAO
Unigene0028400	1744.43	HO1
Unigene0011673	1211.53	HO2
Unigene0044113	950.93	HEMH
Unigene0016337	880.10	NYC1
Unigene0042548	629.87	CHLM
Unigene0042549	443.95	CHLM
Unigene0017708	346.86	CHLD
**Carotenoid metabolism-related genes**		
Unigene0035018	2645.35	CCD4
Unigene0031454	2278.09	CYP707A4
Unigene0041956	2216.15	CYP97A3
Unigene0034645	1699.05	ABA2
Unigene0025400	1501.46	ZDS
Unigene0003112	1016.45	CA2
Unigene0027417	791.28	LCY1
Unigene0012356	713.86	AAO4
Unigene0006524	646.97	VDE1
Unigene0062479	608.21	CCS
Unigene0012498	367.75	CYP707A7
Unigene0008801	334.02	CYP707A4
Unigene0036601	282.07	PSY1
Unigene0011740	152.30	NXS
